# Macrophage ABHD5 promotes colorectal cancer growth by suppressing spermidine production by SRM

**DOI:** 10.1038/ncomms11716

**Published:** 2016-05-18

**Authors:** Hongming Miao, Juanjuan Ou, Yuan Peng, Xuan Zhang, Yujuan Chen, Lijun Hao, Ganfeng Xie, Zhe Wang, Xueli Pang, Zhihua Ruan, Jianjun Li, Liqing Yu, Bingzhong Xue, Hang Shi, Chunmeng Shi, Houjie Liang

**Affiliations:** 1 Department of Oncology, Southwest Hospital, The Third Military Medical University, Chongqing 400038, China; 2 Department of Animal and Avian Sciences, University of Maryland, College Park, Maryland 20742, USA; 3 Department of Biology, Georgia State University, Atlanta, Georgia 30303, USA; 4 Institute of Combined Injury, State Key Laboratory of Trauma, Burns and Combined Injury, Chongqing Engineering Research Center for Nanomedicine, College of Preventive Medicine, Third Military Medical University, Chongqing 400038, China

## Abstract

Metabolic reprogramming in stromal cells plays an essential role in regulating tumour growth. The metabolic activities of tumour-associated macrophages (TAMs) in colorectal cancer (CRC) are incompletely characterized. Here, we identify TAM-derived factors and their roles in the development of CRC. We demonstrate that ABHD5, a lipolytic co-activator, is ectopically expressed in CRC-associated macrophages. We demonstrate *in vitro* and in mouse models that macrophage ABHD5 potentiates growth of CRC cells. Mechanistically, ABHD5 suppresses spermidine synthase (SRM)-dependent spermidine production in macrophages by inhibiting the reactive oxygen species-dependent expression of C/EBPɛ, which activates transcription of the *srm* gene. Notably, macrophage-specific ABHD5 transgene-induced CRC growth in mice can be prevented by an additional SRM transgene in macrophages. Altogether, our results show that the lipolytic factor ABHD5 suppresses SRM-dependent spermidine production in TAMs and potentiates the growth of CRC. The ABHD5/SRM/spermidine axis in TAMs might represent a potential target for therapy.

## Abstract (web summary)

ABHD5 is a co-activator of lipolysis. Here the authors show that in tumour-associated macrophages ABHD5 inhibits ROS-dependent induction of C/EBP_ɛ_, which transcriptionally activates spermidine synthase, and that blocking ABHD5 delays colorectal cancer growth in mice by inhibiting spermidine production.

## Graphical abstract

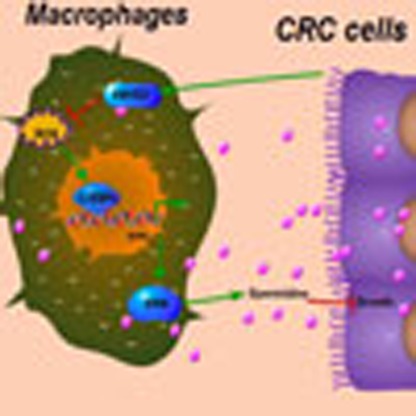

Colorectal cancer (CRC) is the third most common malignancy worldwide^1^. Like many other solid tumours, CRC tumours are infiltrated by a variety of immune cells, including macrophages^2^,^3^. Macrophages are the most prominent innate cells that play a central role in host defence and inflammation^4^. Macrophages that infiltrate tumour tissues are commonly referred to as tumour-associated macrophages (TAMs)^5^,^6^. Animal and clinical studies have demonstrated that TAM-derived tumour-promoting factors are the most important contributors of tumour progress and metastasis^2^,^5^,^6^,^7^,^8^. Among the tumour-promoting factors, inflammatory cytokines are the most widely studied^6^,^7^,^9^, whereas the functional metabolic factors require further characterization.

Recent studies have suggested that metabolic reprogramming in stromal cells plays an essential role in regulating tumour biology^10^,^11^,^12^,^13^. Ovarian cancer induces the transfer of lipids from adipocytes to cancer cells, thereby sustaining tumour growth and metastasis^11^. Reprogramming of glucose and amino acid metabolism in stromal fibroblasts promotes inflammation and tumourigenesis in human prostate cancer^12^. Mesenchymal stem cells induce resistance to chemotherapy through the release of platinum-induced fatty acids^13^. As the most prominent immune cells in the tumour microenvironment, TAMs are loaded with a large number of lipid droplets^14^. However, how the accumulated lipids are catabolized and how TAM metabolism is reprogrammed to adapt the tumour development remains unknown.

AB-hydrolase containing 5 (ABHD5), also known as comparative gene identification-58, is a co-activator of adipose triglyceride lipase (ATGL), a key enzyme involved in lipolysis of triglycerides into diglycerides and free fatty acids^15^. Mutation of ABHD5 in humans causes Chanarin–Dorfman syndrome, which is characterized by ectopic accumulation of neutral lipid in multiple organs or tissues^15^,^16^. Our previous study has demonstrated that ABHD5 acts as an endogenous inhibitor of the NLRP3-inflammasome pathway by suppressing reactive oxygen species (ROS) production in macrophages^17^. We have also found that in CRC cells, ABHD5 expression is decreased and that it functions as a tumour suppressor^18^. Thus, we presumed that ABHD5 in TAMs might be correlated with lipid accumulation and progression of CRC.

This study sought to identify the metabolic activities of TAMs and their roles in the development of CRC. Surprisingly, we found that ABHD5 expression was increased in TAMs in CRC and that macrophage ABHD5 potentiated CRC growth through suppression of spermidine synthase (SRM)-dependent spermidine production in TAMs. Most importantly, we provided evidence that modulation of macrophages to increase spermidine production may be a promising strategy for inhibiting tumour growth.

## Results

### Ectopic expression of ABHD5 in TAMs

**Figure 1:**
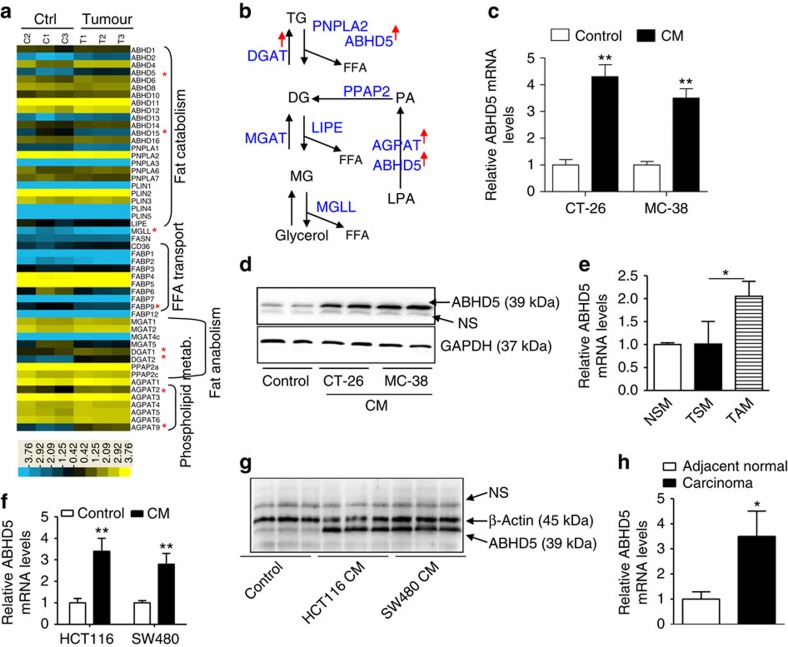
Increased CGI-58 expression in CRC-associated macrophages. (**a**) Murine PMs were treated with regular culture medium (Ctrl group) or co-cultured with CRC cells CT-26 (Tumour group) for 24 h and then subjected to gene-microarray analysis. The expression of key genes involved in fatty-acid transport, fat catabolism, fat anabolism and phospholipid metabolism are displayed in a heat map. *Indicates the expression difference between the Ctrl and Tumour groups (*n*=3, *P<0.05). (**b**) A diagram of glycerolipid metabolism. The red arrow indicates increased expression of genes in TAMs. (**c**) Murine PMs were treated with CM from cultured CRC cells CT-26 or MC-38 for 24 h. Then, the mRNA levels of ABHD5 in macrophages were measured by real-time PCR (*n*=3, **P<0.01). (**d**) Murine PMs were treated as described in **c**, and the cellular ABHD5 protein levels were measured by western blotting. (**e**) Macrophages were isolated from the spleen (TSM) and the tumour tissue (TAM) of CT-26 tumour-bearing mice or from the spleen of normal mice (NSM). The CT-26 cells (5 × 10^6^ cells per mouse) were subcutaneously inoculated at the thighs of BALB/c mice. Ten days later, the tumours and spleens were dissected for macrophage isolation. Then, the ABHD5 mRNA levels in macrophages were measured by real-time PCR (*n*=6–8, *P<0.05). (**f**) mRNA assay of ABHD5 in the murine PMs treated with CM from cultured human CRC cells HCT116 or SW480 for 24 h by real-time PCR (*n*=3, **P<0.01). (**g**) Immunobloting assay of ABHD5 in the murine PMs treated as described in **f**. NS, non-specific band. (**h**) ABHD5 mRNA levels in the macrophages from adjacent normal or carcinoma tissues of human CRC were measured by real-time PCR (*n*=8, *P<0.05). All the histograms in this figure show means±s.e.m., Student's *t*-test.

To investigate how lipid metabolism is reprogrammed in colon-cancer-related TAMs, we performed a gene-microarray analysis to examine the lipid-related gene-expression differences between normal and CRC cell-stimulated peritoneal macrophages (PMs; Fig. 1a). Gene ontology and KEGG analysis indicated that pathways involving with triacylglycerol and phospholipid metabolism were enriched in CRC cell-stimulated PMs (Supplementary Fig. 1a). Among the lipid catabolism-associated genes, ABHD5 expression was markedly upregulated according to the microarray analysis (Fig. 1b). Furthermore, we verified that the mouse CRC cells CT-26 or MC-38-derived conditional medium (CM) stimulated the mRNA (Fig. 1c) and protein (Fig. 1d) levels of ABHD5 in PMs. To verify this finding *in vivo*, we isolated primary macrophages from the spleens of normal mice as well as those from the spleens or tumour tissues of CT-26 tumour-bearing mice. We demonstrated that the mRNA levels of ABHD5 were significantly elevated in the tumour tissue-derived macrophages, in contrast to those in the spleen (Fig. 1e). Furthermore, we confirmed that human CRC cells HCT116 and SW480-derived CM induced ABHD5 mRNA (Fig. 1f) and protein (Fig. 1g) expression in mouse PMs. Finally, we also found that human CRC-tissue-derived macrophages expressed notably higher ABHD5 mRNA levels than those from the corresponding adjacent normal tissues, by real-time PCR (Fig. 1h), and notably higher protein levels (Supplementary Fig. 1b,c), by immunohistochemistry. Thus, ABHD5 might be an essential factor in enhancing lipolysis in lipid-loading TAMs. However, the role of overexpressed macrophage ABHD5 in the development of CRC remains unknown.

### Macrophage ABHD5 stimulates CRC cell growth

**Figure 2:**
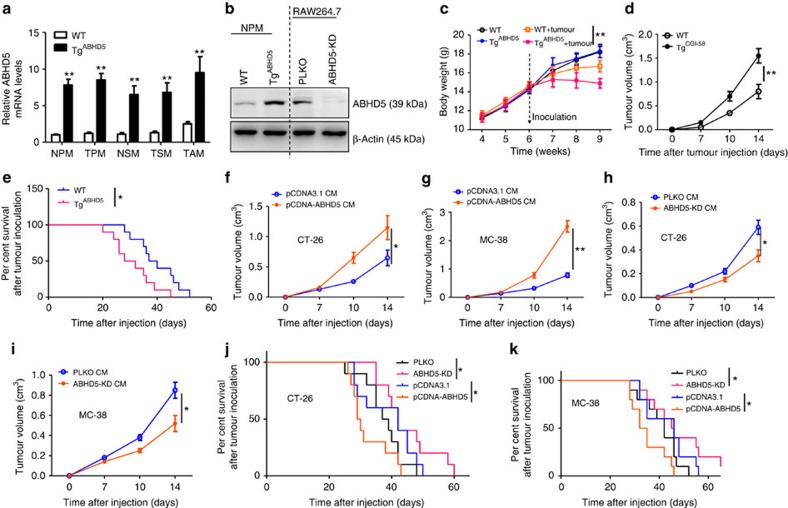
Overexpression of ABHD5 in macrophages potentiates CRC growth in mice. (**a**) ABHD5 mRNA levels increased in different types of macrophages from Tg^ABHD5^ mice versus WT mice. MC-38 tumour cells were subcutaneously injected in the thighs of 6-week-old C57BL/6 mice. Ten days later, PMs of tumour-free mice (NPM) or MC-38 tumour-bearing mice (TPM), spleen macrophages of tumour-free mice (NSM) or MC-38 tumour-bearing mice (TSM) and in MC-38 tumour tissues were collected for mRNA assay with real-time PCR. Histograms show means±s.e.m., (*n*=6, **P<0.01, Student's *t*-test). (**b**) Representative immunoblotting assay of ABHD5 in RAW cells with stable knockdown of ABHD5 (ABHD5-KD) or PLKO vector transfection as a control (PLKO) and primary PMs (NPM) from WT or Tg^ABHD5^ mice. (**c**) The 6-week-old WT and Tg^ABHD5^ mice were subcutaneously inoculated with MC-38 cells, and the body weight was measured weekly. The data represent means±s.e.m., (*n*=6, **P<0.01, Student's *t*-test). (**d**) The 6-week-old WT and Tg^ABHD5^ mice were subcutaneously inoculated with MC-38 cells, and the tumour volume was measured dynamically. The data represent means±s.e.m., (*n*=10, **P<0.01, Student's *t*-test). (**e**) The percentage survival of tumour-bearing mice described in **d**. The data represent means±s.e.m., (*n*=10, *P<0.05, Student's *t*-test). (**f**,**g**) The 6-week-old WT mice were subcutaneously inoculated with CT-26 (**f**) or MC-38 (**g**) cells and treated with CM from pCDNA3.1 or pCDNA-ABHD5-transfected RAW cells. The tumour volume was measured dynamically. The data represent means±s.e.m., (*n*=6, *P<0.05, **P<0.01, Student's *t*-test). (**h**,**i**) The 6-week-old WT mice were subcutaneously inoculated with CT-26 (**h**) or MC-38 (**i**) cells and treated with CM from the control (PLKO) or ABHD5-knockdown (ABHD5-KD) RAW cells. The tumour volume was measured dynamically. The data represent means±s.e.m., (*n*=6, *P<0.05, Student's *t*-test). (**j**,**k**) The percentage survival of CT-26 (**j**) or MC-38 (**k**) tumour-bearing mice treated with different CM from PLKO, ABHD5-KD, pCDNA3.1 or pCDNA-ABHD5 RAW cells. The data represent means±s.e.m., (*n*=10, *P<0.05, Student's *t*-test).

To mimic the ectopic expression of ABHD5 in TAMs and to investigate its role in CRC cell growth *in vivo*, we created a CD11b promoter-driven myeloid-cell-specific ABHD5 transgenic (Tg^ABHD5^) mouse model (Supplementary Fig. 2a), in which the CRC cells were inoculated subcutaneously. ABHD5 mRNA levels were ubiquitously overexpressed in multiple tissue resident macrophages of Tg^ABHD5^ mice compared with wild-type (WT) mice, regardless of tumour inoculation (Fig. 2a). Overexpression of ABHD5 protein was confirmed in the PMs (Fig. 2b), but not in the skins, cerebrums, intestines, livers, epididymal fat pads, colons, hearts and muscles of the Tg^ABHD5^ mice compared with their WT littermates (Supplementary Fig. 2b). After subcutaneous inoculation of MC-38 tumours, the body weights of the Tg^ABHD5^ mice decreased significantly in contrast to those of the tumour-free mice or the tumour-bearing WT mice (Fig. 2c). Moreover, the tumour volumes of the Tg^ABHD5^ mice increased more quickly than those of the WT littermates (Fig. 2d). Accordingly, the tumour-bearing Tg^ABHD5^ mice had a significantly shorter survival time than did the WT mice (Fig. 2e). To further confirm the tumour-promoting role of macrophage ABHD5, the effects of macrophage CM on CRC cells were evaluated. The CM of ABHD5-overexpressing RAW cells notably stimulated the cell viability (Supplementary Fig. 2c,d), cell cycle (Supplementary Fig. 2e), clone formation (Supplementary Fig. 2f) and tumour growth (Fig. 2f,g) of CT-26 and MC-38 cells. Similarly, the CM of ABHD5-knockdown RAW cells significantly attenuated the cell viability (Supplementary Fig. 2g), cell cycle (Supplementary Fig. 2h), clone formation (Supplementary Fig. 2i) and tumour growth (Fig. 2h,i) of CT-26 and MC-38 cells. Finally, we demonstrated that the CM of ABHD5-overexpressing or -knockdown RAW cells suppressed or potentiated the survival time of CT-26 tumour-bearing mice, respectively (Fig. 2j). Identical results were obtained in MC-38-bearing mice (Fig. 2k). Therefore, we concluded that ABHD5-regulated soluble factors derived from macrophages were involved in the regulation of CRC cell growth.

### Macrophage ABHD5 suppresses spermidine production by SRM

**Figure 3:**
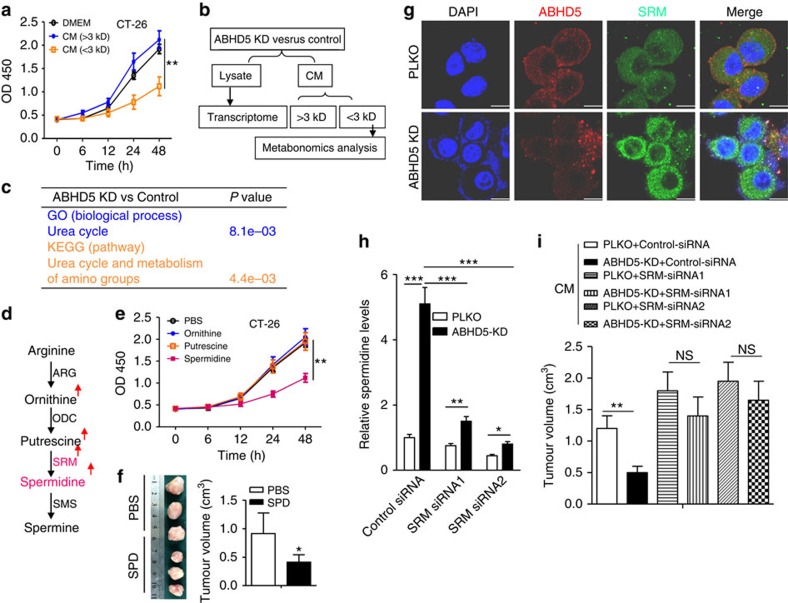
Macrophage ABHD5 potentiates CRC cell growth by suppressing SRM-dependent spermidine production. (**a**) The CM (<3 kD) from ABHD5-KD RAW cells attenuated the growth of CT-26 cells. The CM of ABHD5-KD RAW cells was separated into two fractions (>3 kD and <3 kD). The cell viability of CT-26 cells treated with DMEM, CM (>3 kD) or CM (<3 kD) was measured kinetically. The data represent means±s.e.m., (*n*=5, **P<0.01, Student's *t*-test). (**b**) Technological process for screening ABHD5-mediated metabolic pathway. The control and ABHD5-knockdown RAW cells were subjected to gene expression microarray analysis. The cultured medium (CM) was separated into two fractions (>3 kD and <3 kD). The fraction under 3 kD was subjected to metabonomics analysis. (**c**) The data in **b** were subjected to GO and KEGG analysis. The urea cycle pathway was notably regulated downstream of ABHD5. (**d**) Details of the urea cycle pathway. SRM and spermidine were notably regulated downstream of ABHD5 in RAW cells. The red arrow indicates upregulation. (**e**) The cell viability of CT-26 cells treated with ornithine (2 mM), putrescine (2 mM), spermidine (2 mM) or PBS as a control. The data represent means±s.e.m., (*n*=5, **P<0.01, Student's *t*-test). (**f**) The 6-week-old mice were subcutaneously inoculated with CT-26 tumour and treated with spermidine (2 mM, 100 μl per 2 days) or vehicle control PBS for 2 weeks. Then, the tumour volume was measured. Representative images are displayed. The data represent means±s.e.m., (*n*=5, *P<0.05, Student's *t*-test). (**g**) The PLKO or ABHD5-KD RAW cells were double-stained with ABHD5 (red) and SRM (green) antibody by immunofluorescence. The nucleus was visualized by DAPI (4′, 6-diamidino-2-phenylindole) staining (blue). (**h**) The PLKO or ABHD5-KD RAW cells were transfected with control siRNA (20 nmol ml^−1^), SRM-specific siRNA1 (20 nmol ml^−1^) or siRNA2 (20 nmol ml^−1^), and then the relative spermidine levels in the supernatant were measured. The histograms show means±s.e.m., (*n*=3, *P<0.05, **P<0.01 and ***P<0.005, Student's *t*-test). (**i**) The 6-week-old mice were subcutaneously inoculated with CT-26 tumour cells and treated with the CM as described in **h** for 2 weeks. Then, the tumour volume was measured. The data represent means±s.e.m., (*n*=5, **P<0.01, Student's *t*-test). NS, not significant.

To investigate which soluble factors in the CM of ABHD5-knockdown RAW cells result in the growth inhibition of CRC cells, the CM was separated into two fractions on the basis of size, using a 3 kD cutoff to roughly distinguish between proteins (>3 kD) and metabolites (<3 kD). Only the metabolite fraction (<3 kD CM) exerted an inhibitory role on the growth of CRC cells, whereas the protein fraction mildly stimulated the cell viability (Fig. 3a and Supplementary Fig. 3a). Furthermore, the metabonomics analysis of the CM (<3 kD) was combined with gene expression microarray analysis of the cells to screen the functional metabolic pathway(s) downstream of ABHD5 (Fig. 3b). As previously indicated, gene ontology and KEGG analysis revealed that urea cycle and amino metabolism were regulated by ABHD5 in macrophage-like RAW cells (Fig. 3c). In detail, ABHD5 silence stimulated the expression of SRM as well as the production of ornithine, putrescine and spermidine (Fig. 3d). Furthermore, we observed that spermidine suppressed the growth of CT-26 (Fig. 3e) and MC-38 cells (Supplementary Fig. 3b). However, ornithine and putrescine exerted no significant effects on the growth of CRC cells (Fig. 3e and Supplementary Fig. 3b) or CT-26 tumours (Supplementary Fig. 3c). We also confirmed the inhibitory role of exogenous spermidine on the growth of CT-26 (Fig. 3f) and MC-38 (Supplementary Fig. 3d) cells in the xenograft mouse model.

To further verify the regulatory role of ABHD5 in SRM expression, we performed immunoblotting and immunofluorescence assays, which revealed the inhibitory effect of ABHD5 on SRM expression (Fig. 3g and Supplementary Fig. 3e). However, those two proteins had no direct interaction (Fig. 3g). Interestingly, in contrast to ABHD5 levels, macrophage SRM expression and spermidine levels were decreased in human carcinoma tissues compared with adjacent normal tissues (Supplementary Fig. 3f,g). Similarly, the SRM levels in mouse TAMs decreased relative to those in the spleen (Supplementary Fig. 3h). These results strongly supported the inhibitory effect of ABHD5 on SRM expression.

Therefore, we presumed that macrophage ABHD5 attenuated spermidine production via suppression of SRM. As expected, we demonstrated that knockdown of SRM with siRNAs (Supplementary Fig. 3i) markedly decreased ABHD5 deficiency-induced production of spermidine in RAW cells (Fig. 3h). Similarly, the CM from ABHD5-knockdown RAW cells suppressed CT-26 cell growth, and this effect was rescued by additional knockdown of SRM in ABHD5-deficient RAW cells (Supplementary Fig. 3j). In line with the *in vitro* test results, the xenograft model verified that silence of ABHD5 in RAW cells suppressed CT-26 tumour growth in an SRM-dependent manner (Fig. 3i and Supplementary Fig. 3k).

**Figure 4:**
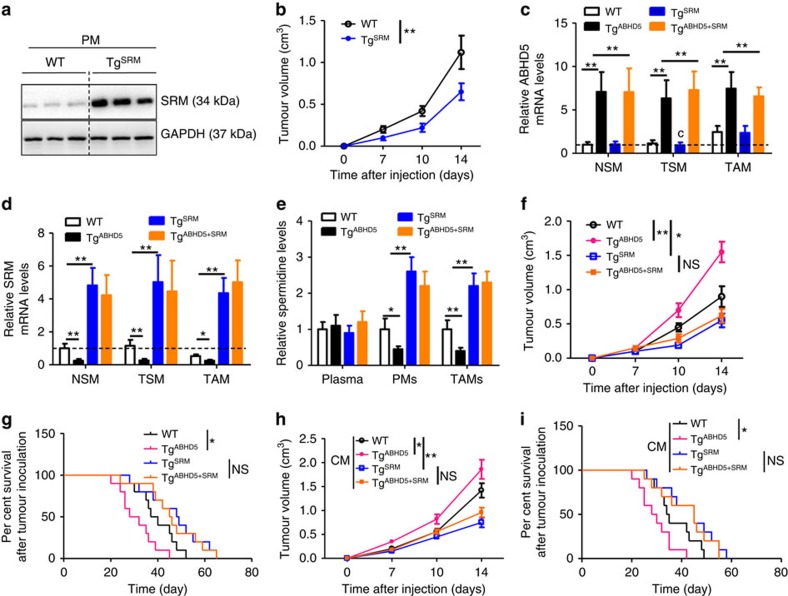
Macrophage ABHD5 potentiates CRC growth by suppressing SRM *in vivo*. (**a**) Immunoblotting assay of SRM in the PMs from WT or Tg^SRM^ mice. (**b**) The 6-week-old WT or Tg^SRM^ mice were subcutaneously inoculated with MC-38 cells, and the tumour volume was measured dynamically. The data represent means±s.e.m., (*n*=8, **P<0.01, Student's *t*-test). (**c**,**d**) Relative mRNA levels of ABHD5 (**c**) and SRM (**d**) in macrophages from the WT, Tg^ABHD5^, Tg^SRM^ or Tg^ABHD5+SRM^ mice. MC-38 tumour cells were subcutaneously injected into the thighs of 6-week-old C57BL/6 mice. Ten days later, tissue macrophages were isolated. NSM, spleen macrophages from tumour-free mice; TSM, spleen macrophages from MC-38 tumour-bearing mice from MC-38 tumour tissues. The data represent means±s.e.m., (*n*=6, *P<0.05, **P<0.01, Student's *t*-test). (**e**) The relative spermidine levels in the plasma, PMs or TAMs from WT, Tg^ABHD5^, Tg^SRM^ or Tg^ABHD5+SRM^ mice that were inoculated with MC-38 tumours subcutaneously for 10 days. The data represent means±s.e.m., (*n*=3, each sample was pooled from 3 individual samples; *P<0.05, **P<0.01, Student's *t*-test). (**f**) The 6-week-old WT, Tg^ABHD5^, Tg^SRM^ and Tg^ABHD5+SRM^ mice were subcutaneously injected with MC-38 cells, and the tumour volume was determined dynamically. The data represent means±s.e.m., (*n*=10, *P<0.05, **P<0.01, Student's *t*-test). (**g**) The percentage survival of WT, Tg^ABHD5^, Tg^SRM^ and Tg^ABHD5+SRM^ mice bearing MC-38 tumours. The data represent means±s.e.m., (*n*=10, *P<0.05, Student's *t*-test). (**h**) The 6-week-old WT BALB/c mice were subcutaneously injected with CT-26 tumours and treated with the CM from PMs of WT, Tg^ABHD5^, Tg^SRM^ and Tg^ABHD5+SRM^ mice. The tumour volume was determined dynamically. The data represent means±s.e.m., (*n*=10, *P<0.05, **P<0.01, Student's *t*-test). (**i**) The percentage survival of subcutaneous CT-26 tumour-bearing BALB/c mice treated with the CM from peritoneal macrophages of WT, Tg^ABHD5^, Tg^SRM^ and Tg^ABHD5+SRM^ mice. The tumour volume was measured dynamically. The data represent means±s.e.m., (*n*=10, *P<0.05, Student's *t*-test). NS, not significant.

To rescue the expression of SRM in macrophages, we constructed a macrophage-specific SRM transgenic (Tg^SRM^) mouse model (Supplementary Fig. 4a,b). The protein levels of SRM were markedly increased in PMs of Tg^SRM^ mice versus WT mice (Fig. 4a). As expected, the growth of subcutaneous MC-38 tumours was significantly suppressed in Tg^SRM^ mice compared with WT mice (Fig. 4b). The inhibitory effects of PM CM from Tg^SRM^ mice on the CRC cell growth were also confirmed by an *in vitro* test (Supplementary Fig. 4c,d).

We further crossed the Tg^SRM^ mice with the Tg^ABHD5^ mice to investigate the reciprocal regulation of those two genes and their roles in CRC cell growth. The mRNA expression of ABHD5 in spleen macrophages and TAMs was increased in Tg^ABHD5^ mice and was not affected by SRM transgenic expression (Fig. 4c). In contrast, Tg^ABHD5^ mice showed decreased SRM expression in spleen macrophages and TAMs, whereas additional transgenic expression of SRM in macrophages rescued this effect (Fig. 4d). In line with the SRM expression, the spermidine levels in the PMs and TAMs from Tg^ABHD5^ mice decreased significantly, and this effect was rescued by macrophage-specific SRM transgenic expression. However, there were no significant differences in plasma spermidine levels among the WT, Tg^ABHD5^, Tg^SRM^ and Tg^ABHD5+SRM^ mice (Fig. 4e). In a cell culture experiment, the PM CM of Tg^ABHD5^ mice potentiated the cell viability of CT-26 (Supplementary Fig. 4e) and MC-38 (Supplementary Fig. 4f), and this effect was antagonized by transgenic SRM expression. In the xenograft model, the MC-38 tumour-bearing mice displayed much larger tumours (Fig. 4f) and shorter survival times (Fig. 4g), whereas additional macrophage-specific SRM transgene abolished this effect. We obtained identical results in CT-26 tumour-bearing mouse model by treating the tumours with different CM from the PMs of WT, Tg^ABHD5^, Tg^SRM^ and Tg^ABHD5+SRM^ mice (Fig. 4h,i).

Interestingly, we also used an inflammation-induced tumourigenesis model to verify the roles of macrophage ABHD5 and SRM in CRC growth. The colitis-associated CRC protocol consisted of an intraperitoneal (i.p.) injection of azoxymethane (AOM), followed by three cycles of dextran sodium sulfate (DSS) administered in the drinking water (Supplementary Fig. 4g). The total numbers of tumours between the WT, Tg^ABHD5^, Tg^SRM^ and Tg^ABHD5+SRM^ mice did not differ significantly, whereas the numbers of tumours with a volume >3 mm^3^ markedly increased in the Tg^ABHD5^ mice versus the WT group, and additional transgenic expression of SRM in macrophages abolished this effect (Supplementary Fig. 4h). These findings strongly indicated that macrophage ABHD5 potentiated CRC cell growth by suppressing SRM-dependent spermidine production, whereas the regulatory effect of ABHD5 on SRM expression remained obscure.

### Macrophage ABHD5 inhibits C/EBPɛ-mediated SRM expression

ABHD5 is a lipolytic co-activator of ATGL^15^. Mutation or loss of function of ABHD5 causes accumulation of neutral lipids in multiple organs and tissues^17^,^19^,^20^,^21^. Herein, we characterized ABHD5 as a suppressor of SRM-dependent spermidine production, through a mechanism that is not fully elucidated. Because ABHD5 is not a transcription factor, we sought to determine whether ABHD5 protein directly interacts with SRM protein in macrophages. Unfortunately, the double-immunofluorescence staining experiment indicated no co-localization of ABHD5 and SRM in RAW cells (Fig. 3g). Thus, we concluded that ABHD5 might regulate SRM expression through an indirect pathway.

**Figure 5:**
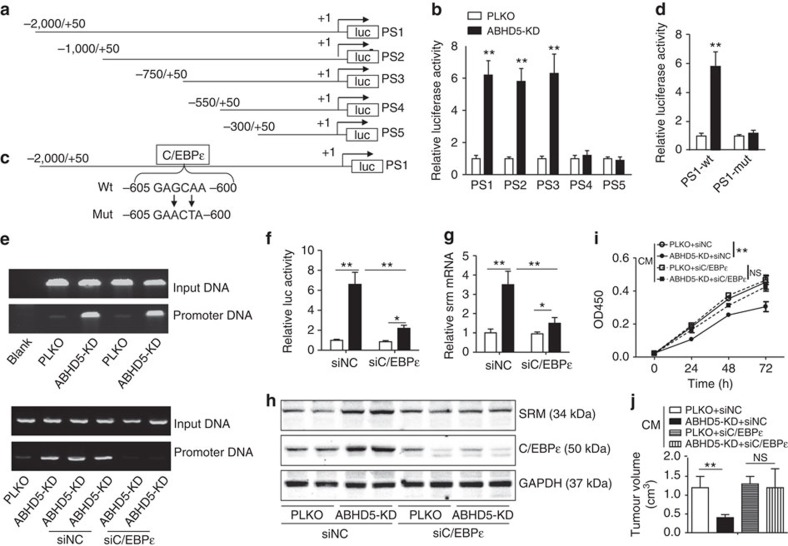
Macrophage ABHD5 suppresses C/EBPɛ-mediated SRM expression. (**a**) A schematic depiction of different mouse SRM promoter regions cloned into the pGL4-basic plasmid. The constructs were designated PS1–PS5. (**b**) Each of the constructs PS1–PS5 (0.4 μg ml^−1^) was transfected into the PLKO or ABHD5-KD RAW cells, and then the luciferase activity was assayed. The data represent means±s.e.m., (*n*=3, **P<0.01, Student's *t*-test). (**c**) A diagram of the PS1 construct with a C/EBPɛ-binding element, which was mutated from 5′-GAGCAA-3′ to 5′-GAACTA-3′ located at −605/−600. (**d**) The construct PS1 (PS1-WT; 0.4 μg ml^−1^) and the C/EBPɛ element-mutated PS1 (PS1-mut) (0.4 μg ml^−1^) were transfected into the PLKO or ABHD5-KD RAW cells for 24 h, and then the reporter-gene assay was performed. The data represent means±s.e.m., (*n*=3, **P<0.01, Student's *t*-test). (**e**) The PLKO or ABHD5-KD RAW cells transfected with control siRNA (siNC) (20 nmol ml^−1^) or C/EBPɛ specific RNA (siC/EBPɛ) (20 nmol ml^−1^) for 24 h were collected for ChIP assay. The interaction between C/EBPɛ protein and SRM promoter DNA was determined. (**f**) The construct PS1 (0.4 μg ml^−1^) and siNC (20 nmol ml^−1^) or siC/EBPɛ (20 nmol ml^−1^) were co-transfected into the PLKO or ABHD5-KD RAW cells for 24 h. Then, the reporter-gene assay was performed. The data represent means±s.e.m., (*n*=3, *P<0.05, **P<0.01, Student's *t*-test). (**g**) SRM mRNA levels of the PLKO or ABHD5-KD RAW cells transfected with siNC (20 nmol ml^−1^) or siC/EBPɛ (20 nmol ml^−1^) for 24 h. The data represent means±s.e.m., (*n*=3, *P<0.05, **P<0.01, Student's *t*-test). (**h**) Immunoblotting assay of SRM and C/EBPɛ in the PLKO or ABHD5-KD RAW cells transfected with siNC (20 nmol ml^−1^) or siC/EBPɛ (20 nmol ml^−1^) for 24 h. (**i**) The cell viability of CT-26 treated with different CM from the PLKO or ABHD5-KD RAW cells, which were transfected with siNC (20 nmol ml^−1^) or siC/EBPɛ (20 nmol ml^−1^) for 24 h. The data represent means±s.e.m., (*n*=5, **P<0.01, Student's *t*-test). (**j**) The 6-week-old WT mice were subcutaneously inoculated with CT-26 cells in the thighs and treated with different CM as described in **i** for 2 weeks. Then, the tumour volumes were measured. The data represent means±s.e.m., (*n*=6, **P<0.01, Student's *t*-test). NS, not significant.

According to the gene expression array results, the mRNA level of SRM was regulated by ABHD5 (Fig. 3d). Therefore, we further determined whether ABHD5 regulates SRM expression at the transcription level. A 2,050-bp (−2,000∼+50) DNA fragment harbouring the mouse SRM promoter was cloned into the pGL4-basic vector (PS1) (Fig. 5a). Furthermore, a series of mutated plasmids containing different regions of SRM promoter (PV2–PV5) were constructed (Fig. 5a). Reporter-gene assays indicated that the promoter region −750∼−550 bps was required for ABHD5 deficiency-induced SRM transcriptional activity (Fig. 5b). Interestingly, the sequences −716∼−423 bps in the mouse SRM gene were highly conserved (84% similarity), as compared with the sequences −990∼−705 bps in the human SRM gene, according to DNA sequence alignment (Supplementary Fig. 5a,b). Thus, we expected important *cis*-acting elements of the *srm* gene to be located in this region. To identify the transcriptional factors involved in ABHD5 deficiency-induced SRM transcription, we predicted the putative transcription factor binding sites in DNA sequences from −716 to −423 bp in the mouse SRM gene by using online software (http://alggen.Isi.upc.es). Several potential elements for STAT5A, Elk-1 and C/EBPɛ (−605∼−600) had high scores. To determine whether those elements are functional in ABHD5 deficiency-stimulated SRM expression, we performed site-directed mutagenesis of those elements. Reporter-gene assay experiments verified that only the core sequences of C/EBPɛ-binding element (5′-GAGCAA-3′) were required for ABHD5 silence-stimulated SRM promoter activity (Fig. 5c,d), whereas the binding elements of STAT5A and Elk-1 were not functional (data not shown). Similarly, a conserved and functional C/EBPɛ-binding element (5′-GAGCAA-3′) located at −778∼−773 bps was identified in the human *srm* gene promoter (Supplementary Fig. 5c–e).

To further verify the direct interaction between C/EBPɛ protein and the predicted binding site in the mouse *srm* gene promoter, chromatin immunoprecipitation (ChIP) assays were performed. As shown in Fig. 5e, C/EBPɛ protein directly bound the predicted elements locating at −605∼−600 bps. The binding activity was stimulated by ABHD5 silencing, and siRNA-mediated C/EBPɛ knockdown (Supplementary Fig. 5c) blocked this effect. Similarly, ABHD5 deficiency-induced SRM promoter activity (Fig. 5f) and changes in mRNA (Fig. 5g) and protein (Fig. 5h) levels was largely prevented by additional silencing of C/EBPɛ in RAW cells. Furthermore, we examined the function of C/EBPɛ in regulating macrophage ABHD5-mediated CRC cell growth. As expected, ABHD5 deficiency-suppressed CRC cell proliferation (Fig. 5i) and tumour growth (Fig. 5j) were rescued by additional C/EBPɛ knockdown in RAW cells. These data indicated that ABHD5 suppresses C/EBPɛ-mediated SRM expression in macrophage-like RAW cells. However, the mechanism linking ABHD5 to C/EBPɛ repression requires further elucidation.

### ABHD5 deficiency-induced ROS stimulate C/EBPɛ expression

**Figure 6:**
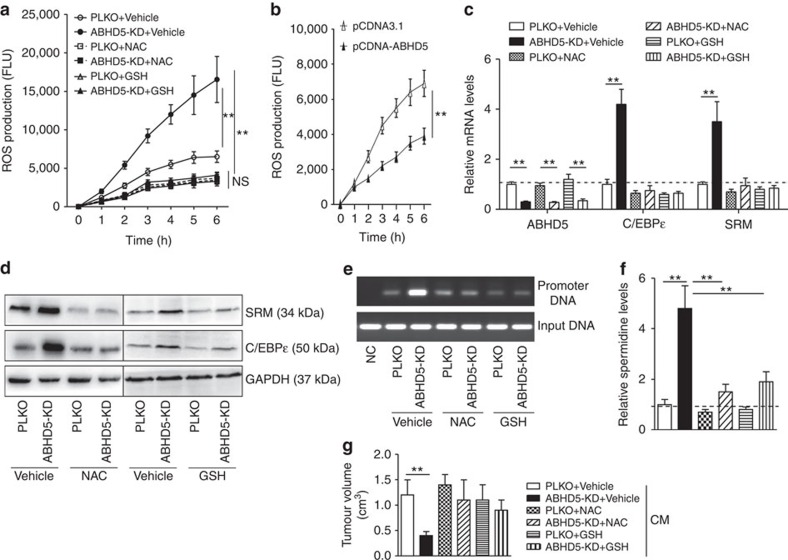
Macrophage ABHD5 suppresses ROS-dependent C/EBPɛ expression. (**a**) The ROS production in the PLKO or ABHD5-KD RAW cells treated with NAC (20 mM), GSH (10 mM) or vehicle control PBS for 24 h were measured. The data represent means±s.e.m., (*n*=5, **P<0.01, Student's *t*-test). (**b**) The ROS production in the pCDNA3.1 or pCDNA-ABHD5-transfected RAW cells. The data represent means±s.e.m., (*n*=5, **P<0.01, Student's *t*-test). (**c**) ABHD5, C/EBPɛ and SRM mRNA levels in the RAW cells as described in **a**. The data represent means±s.e.m., (*n*=4, **P<0.01, Student's *t*-test). (**d**) Immunoblotting assay of C/EBPɛ and SRM in the RAW cells described in **a**. (**e**) The ChIP assay was performed to determine the interaction between C/EBPɛ protein and SRM promoter in the RAW cells described in **a**. (**f**) Supernatant spermidine levels in the RAW cells described in **a**. The data represent means±s.e.m., (*n*=4, **P<0.01, Student's *t*-test). (**g**) Tumour volumes of the mice that were subcutaneously inoculated with CT-26 cells in the thighs and treated with the CM from the RAW cells described in **a** for 2 weeks. The data represent means±s.e.m., (*n*=6, **P<0.01, Student's *t-*test). NS, not significant.

C/EBPɛ, a member of the CCAAT/enhancer-binding proteins, is a myeloid-specific activator of cytokines and chemokines that regulates differentiation and inflammatory response of macrophages^22^,^23^. The expression of CCAAT/enhancer-binding proteins is tightly regulated by LPS, NF-κB or cytokine (IL-1β, and so on) signals in macrophages^24^. We have recently demonstrated that macrophage ABHD5 deficiency induces ROS-dependent IL-1β production^17^,^25^. Therefore, we presumed that ABHD5 might suppress C/EBPɛ expression via inhibition of ROS. Consistently with the results of the previous studies, silencing of ABHD5 resulted in ROS accumulation in RAW cells (Fig. 6a), and overexpression of ABHD5 had the opposite effect (Fig. 6b). Antioxidant treatment with NAC or GSH prevented ABHD5 deficiency-stimulated ROS production (Fig. 6a). Similarly, silencing of ABHD5-induced mRNA (Fig. 6c) and protein (Fig. 6d) expression of C/EBPɛ and SRM, whereas antioxidant treatment blocked this effect (Fig. 6c,d). The ChIP assay also verified that antioxidant treatment decreased the ABHD5 deficiency-induced binding activity between C/EBPɛ protein and SRM promoter DNA in RAW cells (Fig. 6e). Furthermore, we demonstrated that RAW cell ABHD5 silence-potentiated spermidine production was decreased by antioxidant treatment with NAC or GSH (Fig. 6f). Finally, we verified that RAW cell ABHD5 silence-suppressed CRC growth was rescued by antioxidant treatment (Fig. 6g). Thus, we identified a novel pathway (ABHD5-ROS-C/EBPɛ) in macrophage-like RAW cells and revealed its role in regulating CRC growth.

Our previous study has demonstrated that ABHD5 is deficient in colorectal carcinoma versus adjacent normal tissues and functions as a tumour suppressor^18^. Hence, deficiency of ABHD5 in colon tissues would be expected to potentiate spermidine production and subsequently suppress tumour growth, seemingly contradicting ABHD5's function as a tumour suppressor. Indeed, C/EBPɛ was expressed only in myeloid cells but not in CRC cells (Supplementary Fig. 6a), consistently with previous reports^22^,^23^. Therefore, ABHD5 deficiency in CRC cells did not stimulate SRM expression in the mRNA (Supplementary Fig. 6b) or protein (Supplementary Fig. 6c) levels. In line with the SRM expression, ABHD5 did not regulate spermidine production in CRC cells (Supplementary Fig. 6d). However, when we overexpressed SRM in CRC cells CT-26 or MC-38 (Supplementary Fig. 6e), spermidine production was largely increased (Supplementary Fig. 6f). Consequently, the CT-26 or MC-38-inoculated tumours were inhibited by SRM overexpression (Supplementary Fig. 6g,h).

## Discussion

**Figure 7:**
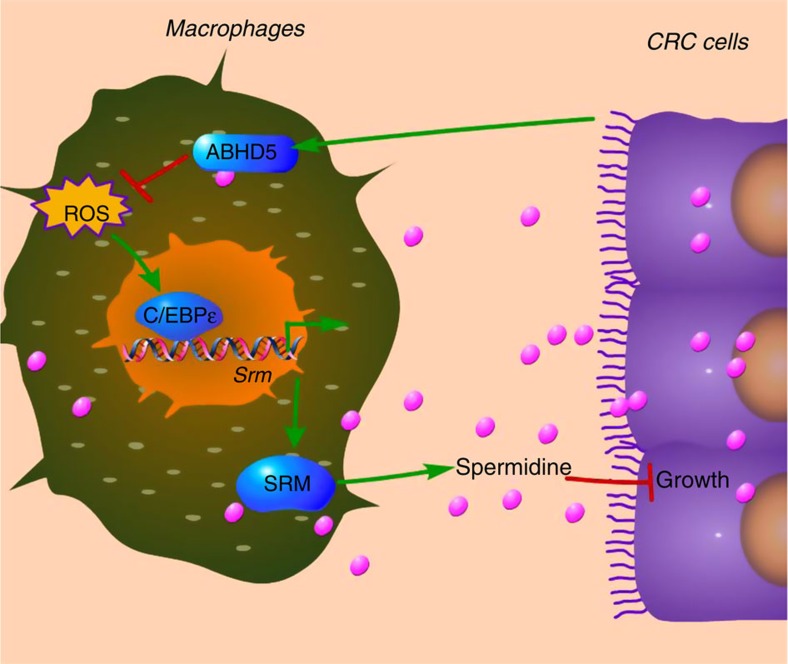
Proposed hypothesis for macrophage ABHD5-spermidine axis in regulating CRC cell growth. CRC cells reprogram TAMs by upregulating ABHD5 expression. Macrophage ABHD5 suppresses ROS accumulation and C/EBPɛ-mediated transcription of SRM to inhibit spermidine production. The macrophage-derived spermidines exert an inhibitory effect on the growth of CRC cells. The arrow indicates positive regulation, and the line represents negative regulation.

Our findings suggest a novel mechanism linking lipid and polyamine metabolism in TAMs to the growth of CRC. We revealed that ABHD5 expression was increased in TAMs. The increased ABHD5 in macrophages suppressed ROS accumulation, inhibited C/EBPɛ-dependent SRM/spermidine production and facilitated the growth of CRC (Fig. 7).

The well-established function of ABHD5 is a co-activation of ATGL in triglyceride hydrolysis^15^. Mutation or loss function of ABHD5 causes neutral lipid accumulation in multiple organs or tissues^16^,^17^,^21^. In this study, we characterized ABHD5 as a regulator of polyamine metabolism. In particular, ABHD5 suppressed spermidine production by inhibiting the expression of SRM. A recent report has claimed that ABHD5 protein directly interacts with SRM-1 in *Arabidopsis thaliana* plants, thereby regulating polyamine metabolism^26^. However, this phenomenon did not exist in mouse macrophages according to the results of the present study. Through reporter-gene assays, we identified C/EBPɛ as a novel transcriptional factor of SRM. Thus, we concluded that ABHD5 suppressed SRM expression via C/EBPɛ. We are also the first to report that C/EBPɛ is suppressed by ABHD5-inhibited ROS production. We presumed that macrophage ABHD5-mediated mitochondrial function might be involved in this mechanism because mitochondrial dysfunction induces ROS accumulation in ABHD5-deficient macrophages^17^.

Metabolic reprogramming, which provides tumour cells with essential substrates and energy, is an important feature of tumour progression^27^,^28^,^29^,^30^. The metabolic changes in the tumour microenvironment include metabolic reprogramming in stromal cells and tumour cells. In this study, we uncovered multiple reprogrammed metabolic pathways in TAMs and their roles in the development of CRC.

First, we initiated our study by investigating lipid metabolism in TAMs. It has been well-documented that tumour-associated myeloid cells are rich in lipid droplets^14^. How those lipids are catabolized to fuel the TAMs is a very interesting question. Among the catabolic enzymes of glycerolipids^31^, only ABHD5 was found to be increased in TAMs of CRC. We have previously demonstrated that ABHD5 supports mitochondrial function in macrophages. Moreover, macrophage ABHD5 is required to sustain the M2 phenotype^17^,^32^,^33^, which contributes to the progression of tumours^5^. Thus, we conclude that TAMs are reprogrammed to serve the development of CRC through ectopic expression of ABHD5, without which the TAMs might not survive because of a deficiency of fatty-acid oxidation and energy production.

Second, we focused on the activated polyamine pathway in TAMs and its role in the growth of CRC. Interestingly, SRM was negatively regulated by ABHD5, a well-documented activator of lipolysis. Thus, ABHD5 stands at the crossroads of lipid catabolism and polyamine synthesis, thus further highlighting the role of ABHD5 in metabolic regulation.

The polyamines spermidine, spermine, putrescine and cadaverine are an essential class of metabolites found throughout all kingdoms of life^34^. Published data on the role of polyamines in tumour biology remain controversial. Previous studies have mainly focused on the whole-polyamine pathway by activating/silencing ornithine decarboxylase, a key enzyme upstream of the polyamine synthesis pathway^35^,^36^, or spermidine/spermine acetyltransferase, a key enzyme in polyamine catabolism^37^,^38^,^39^. Poulin *et al.*^40^ have claimed that excessive polyamine accumulation induces cell apoptosis in ornithine decarboxylase-overproducing L1210 cells. Despite the strong association of polyamines with cancer, to date, attempts to use this pathway as a therapeutic target have failed^34^. It should be noted that each member of the polyamines might have a unique role in different normal or cancer cells. Therefore, according to the seemingly ‘contradictory' findings, we suggest that a more-specific activator or inhibitor should be used to interrupt polyamine metabolism in tumour treatment. Our study suggests that drugs specific to SRM or spermidine might be useful in the treatment of CRC.

By investigating the interaction between macrophages and CRC cells, we identified multiple pathways that might be applicable in the arrangement of tumour treatment. (1) Pro-oxidative treatments such as radiotherapy might potentiate the ROS-C/EBPɛ-SRM-spermidine pathway in macrophages, thereby impairing CRC growth. (2) Metabolic intervention of specific polyamines such as spermidine might affect tumour cell growth. (3) Notably, ABHD5 might not be easily targeted in the treatment of CRC because of a deficiency of ABHD5 in cancer-cell-potentiated metastasis^18^, whereas overexpression of ABHD5 in TAMs promoted tumour growth in the present study. All the aforementioned metabolic strategies might be used in combination with existing chemotherapy regimens.

It is very interesting that ABHD5 showed opposing functions in CRCs and TAMs in our studies. To the best of our knowledge, ABHD5 also shows opposing functions under the same microenvironment in obesity research. For example, a deficiency of ABHD5 in macrophages potentiates high-fat diet-induced chronic inflammation and insulin resistance^17^. However, transgenic expression of ABHD5 in adipose tissues or liver-specific knockout of ABHD5 has no effect on systemic insulin sensitivity in response to a high-fat diet^21^,^41^. Interestingly, specific deletion of ABHD5 in muscle improves insulin sensitivity in mice on a high-fat diet^19^. Therefore, the aforementioned studies indicate a tissue/cell-specific role of ABHD5. Similarly, in our studies on CRC, we have demonstrated that ABHD5 deficiency promotes colorectal tumour development by inducing glycolysis and epithelial-mesenchymal transition^18^. In this study, we demonstrated that ABHD5 was overexpressed in CRC-associated macrophages and potentiated the growth of CRC cells by regulating spermidine metabolism in macrophages. Clearly, ABHD5 in different cells exerts different functions through different mechanisms.

Another interesting phenomenon was that the expression of ABHD5 in CRC cells or cancer-associated macrophages was opposite. To the best of our knowledge, this phenomenon also occurs for other genes. We found that another lipolysis enzyme MGLL, which is overexpressed in CRC cells^42^, was also decreased in CRC-associated macrophages (Fig. 1a). In addition, we observed that cancer-associated T cells overexpressed ABHD5 (data not shown). We presumed that the tumour cells and the associated stromal cells (for example, macrophages, T cells) under the same microenvironment might exhibit opposite expression of certain genes, particularly metabolism-related genes. The tumour microenvironment is innutritious and tumour cells compete with stromal cells for important nutrients, such as glucose, fatty acids and amino acids^11^,^43^,^44^. Generally, cancer cells are dominant in the nutrient competition. Thus, stromal cells lack some nutrients, and metabolic pathways might be modified to support cell survival. Therefore, relative to cancer cells, stromal cells (for example, macrophages, T cells) might have a different signature of metabolic genes. However, the precise regulatory mechanisms of gene expression in the tumour microenvironment require further exploration in future studies.

In conclusion, our findings provide the first evidence that ABHD5 expression is increased in TAMs. Macrophage ABHD5 suppresses C/EBPɛ-dependent SRM expression, thus inhibiting spermidine production and subsequently removing the inhibitory effects of TAM-derived spermidine on CRC growth. Our findings provide a novel mechanism linking a lipolytic factor to polyamine metabolism in TAMs and the growth of CRC, and shed light on therapeutic strategies to treat this malignancy.

## Methods

### Generation of macrophage-specific transgenic mice

The cDNA of mouse *abhd5* was subcloned into a construct containing the human CD11b promoter to drive macrophage-specific gene expression^45^,^46^. The macrophage-specific *abhd5* transgenic construct was microinjected into C57BL/6 embryos according to standard protocols, and the founders were crossed with the WT C57BL/6 strain. The line with the highest level of ABHD5 expression in macrophages (Tg^ABHD5^) was selected for further study. With the same protocol, the cDNA of mouse *srm* was subcloned and macrophage-specific *srm* transgenic (Tg^SRM^) mice were obtained. To rescue the SRM expression in the macrophages of Tg^ABHD5^ mice, Tg^ABHD5^ mice were mated with Tg^SRM^ mice to obtain the double-transgenic (Tg^ABHD5+ SRM^) mice. This study was approved by the Institutional Animal Care and Use Committee of Third Military Medical University and was carried out in accordance with relevant guidelines.

### Subcutaneous xenograft models

Six-week-old C57BL/6 or BALB/c mice (body weight: 17–19 g) were purchased from the Experimental Animal Center at the Third Military Medical University. The BALB/c mice were subcutaneously injected with CT-26 CRC cells (5 × 10^6^ cells per mouse), and the C57BL/6 mice were inoculated with MC-38 CRC cells (5 × 10^6^ cells per mouse) in their thighs. The CM or the drugs (100 μl) were administrated subcutaneously around the tumour basement and were provided once every 2 days. The mice were killed 2 weeks after injection, and the tumours were dissected. The tumour volume (size) was calculated as 0.523 × (length × width × height). This study was approved by the Institutional Animal Care and Use Committee of the Third Military Medical University and was carried out in accordance with relevant guidelines.

### Chemical-induced model of CRC

The colitis-associated CRC protocol consists of an i.p. injection of AOM, followed by three cycles of DSS administered in drinking water. In detail, the 6-week-old male transgenic or WT C57BL/6 mice were subjected to i.p. injection of AOM (10 mg kg^−1^) once in the first week, and this was followed by the administration of 2% DSS-containing drinking water for 1 week and normal drinking water for 2 weeks. The abovementioned cycles of drinking water administration were repeated three times. Then, the mice were killed. The tumour numbers and volumes were evaluated. This study was approved by the Institutional Animal Care and Use Committee of the Third Military Medical University and was carried out in accordance with relevant guidelines.

### Cell culture

The Raw264.7 macrophage-like cell line (RAW cell) and CT-26, HCT116 and SW480 cells were purchased from ATCC (Rockville, MD, USA). MC-38 cells were provided by JENNIO Biological Technology (Guangzhou, China). All cells had been authenticated and tested for mycoplasma. All the cell lines and primary mouse macrophages were grown in DMEM or 1640 medium supplemented with 10% fetal bovine serum (FBS) at 37 °C in a humidified 5% CO_2_ atmosphere.

### Preparation of CM

The PMs or RAW cells with different gene functions were cultured in 250 ml flasks in regular medium (DMEM supplemented with 10% FBS). At the time of 80% confluence, 10 ml of DMEM with 1% FBS and different drugs was added to each flask and was re-collected 48 h later to obtain macrophage-primed medium. The CM was obtained by mixing the macrophage-primed medium with the regular medium (v/v=1:1). The CM was used to treat the cultured CRC cells *in vitro* or xenografts *in vivo*.

### Isolation of PMs

Each mouse was injected (i.p.) with 2 ml of 3% thioglycollate (#T9032, Sigma) on day 1 and killed with isoflurane on day 3. After i.p. injection of 5 ml DMEM cell culture medium containing 10% FBS, as well as penicillin and streptomycin, the peritoneal cells were collected in cell culture dishes. Two hours later, the floating cells were removed by washing the cells with phosphate-buffered saline (PBS). The attached cells were considered to be PMs (purity: ∼90%) and were subjected to further experiments.

### Isolation of macrophages from spleens or tumour tissues

Mouse spleens were dissected and placed in fresh Buffer A containing PBS, 2 mM EDTA, and 0.5% bovine serum albumin in a 60-mm dish. The spleens were gently rubbed between the two rough sides of frosted sides (#12-550-34, Fisher). The dissociated cells were collected in a 15-ml tube and centrifuged at 400*g* for 5 min. The pellets were re-suspended in 5 ml ACK lysing buffer (#10-548E, Lonza), kept still for 5 min at room temperature, diluted to 15 ml with 10 ml DMEM and centrifuged at 1,000*g* for 5 min. The ACK lysing buffer was then aspirated, and 1 ml Buffer A was added to resuspend the pellets. The re-suspended cells were filtered through a 100-μm filter (#08-771-19, Fisher) and spun at 400*g* for 5 min. These cells were washed with Buffer A again and prepared for macrophage isolation.

The fresh CRC tissues were cut into pieces and digested in collagenase B (1 mg ml^−1^, #11088807001, Roche) containing Buffer A. The dissociated cells were collected into a 15-ml tube and centrifuged at 400*g* for 5 min. The pellets were re-suspended with ACK lysing buffer and washed with Buffer A before filtration with a 100-μm filter. These cells were collected for further macrophage isolation.

Isolation of macrophages (F4/80+ cells) from splenocytes or CRC-tissue-derived cells was performed by magnetic immunoaffinity isolation with anti-F4/80 antibodies conjugated to magnetic beads (MACS; Miltenyi Biotec). Cells were isolated using positive-selection columns (MACS) before the preparation of whole-cell lysates for mRNA analysis by real-time PCR. The purity of the MACS-isolated macrophages was high, reaching up to 95%.

### Establishment of stable cell lines

ABHD5-KD RAW cells were previously established with stable knockdown of murine ABHD5 expression by shRNA, and the control cells were PLKO-transfected RAW cells^17^. pCDNA-ABHD5 RAW cells were established with stable overexpression of murine ABHD5, and the control cells were pCDNA3.1-transfected macrophages^25^.

### Cloning of reporter genes

The DNA fragments of the mouse SRM promoter fusion reporter constructs shown in Fig. 5a were generated from RAW cell genomic DNA by PCR amplification using the KOD-Plus Kit (#KOD-201, Toyobo). The primers used for DNA fragment amplification were as follows: −2,000/+50F: 5′-cAAGCTTtttccagtatcggaatct-3′; −1,000/+50F: 5′-cAAGCTTactcaggacctttggaag-3′; −750/+50 F: 5′-cAAGCTTccttcagctgtgcgttat-3′; −550/+50F: 5′-cAAGCTTttccttgacattttcata-3′; −300/+50F: 5′-cAAGCTTgcccagactagacacgat-3′. The reverse primer for all the fragments was 5′-aCTCGAGcggatggcggcggggcc-3′. The restriction enzymes used for the cloning of fusion reporter constructs included *Hin*dIII (5′-AAGCTT-3′) and *Xho*I (5′-CTCGAG-3′).

### Site-directed reporter-gene mutation

The potential C/EBPɛ-binding site (5′-GAGCAA-3′) in the SRM promoter region was mutated as 5′-GAACTA-3′ with a MutanBEST Kit (#401, Takara Bio., Japan). The primer sequences are available on request. PCR was performed using *pyrobest* DNA polymerase, followed by blunting, kination and ligation. The mutant plasmid was transformed into *Escherichia coli* DH5α, and the positive clone was selected and confirmed by DNA sequencing.

### Reporter-gene assays

Transfection was performed following the protocol of Lipofectamine-2000 (#12566014, Invitrogen). The ratio of liposome to DNA was 2:1 (μl μg^−1^). The concentration for RNA transfection was 20 nmol ml^−1^, and for reporter-gene transfection it was 0.4 μg ml^−1^. The reporter genes were transfected into RAW cells cultured in 96-well plates, and the luciferase activities of the cell lysates were evaluated according to the manufacturer's instructions (#E1910, Promega). The total protein concentration in each assay was measured as an internal control.

### ChIP assay

This experiment was performed to measure ABHD5-mediated binding between C/EBPɛ protein and SRM promoter DNA. Briefly, cultured RAW cells (1 × 10^6^) were cross-linked with 1% formaldehyde, and this was followed by sonication. Supernatants with equal amounts of protein were immunoprecipitated with 1 μg of mouse C/EBPɛ antibody (2 ng μl^−1^) or rabbit IgG (2 ng μl^−1^) as a control using a ChIP Kit (#17-10460, Millipore Corp.) according to the manufacturer's protocol. The immunoprecipitates were analysed by PCR to detect the co-immunoprecipitated DNA containing the functional C/EBPɛ-binding site. The primers were designed as follows: forward: 5′-ttattcgtggttcgtcttcc-3′, reverse: 5′-cttatcagaatcacattaag-3′. The length of the desired product was 100 bp.

### Transient gene overexpression or silence

For transient silencing of mouse SRM, the siRNAs specific to the murine *srm* gene (siRNA1 S: 5′-GGCGAUGGCUUUGAGUUCAtt-3′; siRNA1 A: 5′-UGAACUCAAAGCCAUCGCCtt-3′; siRNA2 S: 5′-UGCACCUGGACCUCAUCAAtt-3′; siRNA2 A: 5′-UUGAUGAGGUCCAGGUGCAtt-3′) and the scrambled control siRNA (5′-UUCUCCGAACGUGUCACGUtt-3′) were synthesized and transfected (20 nmol ml^−1^) into the macrophages for >24 h. For knockdown of mouse C/EBPɛ, the siRNAs specific to the murine *c/ebpɛ* gene (siRNA S: 5′-UAGAUUUGCCCUAGGUUUGCCtt-3′; siRNA A: 5′-CAAACCUAGGGCAAAUCUAGGtt-3′) were used. For overexpression of C/EBPɛ in macrophages, the reconstructed pCDNA3.1 vector-based murine C/EBPɛ (cloning from #MC212232, Origene) overexpression plasmids (0.4 μg ml^−1^) were transfected for >24 h.

### Cell-viability assay

The CCK8 assay was performed according to the manufacturer's protocol (C0037, Beyotime, China). CT-26 or MC-38 cells (2,000 cells per 100 μl medium) were plated in 96-well plates. Twelve hours later, the cells were treated with different CMs. At different time points, 10 μl of CCK8 solution was added to each well, and the wells were cultured at 37 °C in a humidified 5% CO_2_ atmosphere for 1 h. Then, the absorbance at 450 nm of each well was measured.

### Cell cycle analysis

Cell cycle analysis was performed according to the manufacturer's protocol (C1052, Beyotime, China). Briefly, the CT-26 or MC-38 cells were digested to single cells and washed twice with cold PBS. Then, the cells were fixed with cold ethanol (70%) for 12 h and washed with cold PBS. Finally, the cells were stained with propidium iodide for 30 min before flow cytometry analysis.

### Clonogenic assay

CT-26 or MC-38 CRC cells were plated into 6-well plates. Ten days after plating, the colonies were stained with a crystal violet solution and counted. The colony-formation efficiency was calculated as follows: the number of colonies/the number of plated cells.

### Flow cytometry assay of apoptosis

Cell apoptosis was assessed with an Annexin V-FITC Apoptosis Analysis Kit (AO2001-02A-H, Sungene Biotech, China). After collecting, the cells were washed twice with cold PBS and re-suspended in 100 μl of 1 × Annexin binding buffer. Five microliters Annexin V-FITC and 5 μl 7-AAD solutions were then added to the cell suspension and incubated at 37 °C for 15 min. The stained cells were analysed with a FACS system (FACSAria, BD Bioscience).

### Metabonomics analysis

The macrophage supernatant or plasma samples were subjected to metabolic analysis by gas chromatography and time-of-flight mass spectrometry with an instrument developed by the BioTree Company (Shanghai, China). The production of metabolites was reported in terms of relative values.

### Assays of ROS in cultured RAW cells

The cells were incubated in 96-well plates and treated with 5-(and-6)-chloromethyl-2′,7′-dichlorodihrofluorescein diacetate acetylester (DCFDA, 50 μM; #D6883, Sigma-Aldrich) for 6 h. ROS production was determined by hydrolysis of DCFDA to fluorescent 2′,7′-dichlorofluorescein, which is generally exerted by several reactive radical species and allows for the assessment of general oxidative stress. DCFDA conversion was kinetically measured every 1 h in a Microplate Reader at excitation and emission wavelengths of 488 and 535 nm, respectively.

### Real-time PCR

Total RNAs were isolated using a peqGold Total RNA Kit including DNase digestion (Peqlab, Erlangen, Germany). RNAs were transcribed into cDNAs using Omniscript (Qiagen, Hilden, Germany). qPCR was performed using the 7900HT Fast Real-Time PCR system (Applied Biosystems, Darmstadt, Germany). Expression levels were normalized to β-actin. Reactions were performed in duplicate using Applied Biosystems Taqman Gene Expression Assays and Universal PCR Master Mix. The relative expression was calculated by the 2(^**–DDCt**^) method. The primers are available on request.

### Western blot

Tissue and cell proteins were extracted with RIPA Lysis Buffer (#P0013, Beyotime, China) and quantified with a BCA kit (#P0009, Beyotime, China). Fifty micrograms of each protein sample was separated by 8 or 10% SDS-PAGE and transferred to a polyvinylidene-difluoride membrane. The membranes were blocked with 5% non-fat milk and incubated with primary antibodies for 10 h at 4 °C. The membranes were rinsed 5 times with PBS containing 0.1% Tween 20 and incubated for 1 h with the appropriate horseradish peroxidase-conjugated secondary antibody at 37 °C. Membranes were extensively washed with PBS containing 0.1% Tween 20 3 times. The signals were stimulated with Enhanced Chemiluminescence Substrate (#NEL105001 EA, PerkinElmer) for 1 min and detected with a Bio-Rad ChemiDoc MP System (170-8280). The primary antibodies included anti-ABHD5 (#H00051099-M01, Abnova; the dilution ratio was 1:1,000), anti-GAPDH (#2118, Cell signaling; the dilution ratio was 1:2,000), anti-β-actin (#3700, Cell signaling; the dilution ratio was 1:2,000), anti-Tubulin (#2148, Cell Signaling, the dilution ratio was 1:2,000), anti-C/EBPɛ (#25770, Santa Cruz; the dilution ratio was 1:1,000) and anti-SRM (#19858-1-AP, Proteintech; the dilution ratio was 1:1,000). Images were cropped for presentation. Full-size images are presented in Supplementary Figs 7–13.

### Immunofluorescence staining

The cells on the coverslips were fixed in 4% ice-cold paraformaldehyde in PBS for 20 min, washed with PBS 3 times (5 min each), and incubated for 30 min at room temperature in a protein-blocking solution. The coverslips were incubated with the primary antibodies (anti-ABHD5, #PAB12500, Abnova, the dilution ratio was 1:1,000; anti-SRM, #19858-1-AP, Proteintech, the dilution ratio was 1:1,000) for 1 h at 37 °C and then at 4 °C overnight. After being washed, the coverslips were incubated at 37 °C for 1 h with TRITC-conjugated goat anti-rabbit IgG (1:50, Beyotime, China). The cells were counterstained with 4′,6-diamidino-2-phenylindole to reveal cell nuclei. The specificity of the primary antibody was verified by omitting that antibody in the reaction.

### Immunohistochemistry of patient samples

Formalin-fixed and paraffin-embedded CRC samples in this study were obtained from the tissue bank of the Department of Oncology at Southwest Hospital at the Third Military Medical University. All tumours were primary and untreated before surgery, and the specimens were anonymized. Tumour tissues were collected in compliance with the regulations approved by the Scientific Investigation Board of the hospital. All tissue slides were de-waxed and rehydrated. The slides were then incubated in 0.3% H_2_O_2_ in methanol for 30 min to block endogenous peroxidase activity. Antigens were retrieved with 10 mmol l^−1^ sodium citrate (pH 6) for 5 min in a pressure cooker. The slides were then incubated with the selected antibodies (anti-CD68, NB100-683, Novus Biologicals, the dilution ratio was 1:1,000; anti-ABHD5, #PAB12500, Abnova, the dilution ratio was 1:1,000) at 4 °C overnight. The slides without treatment of the primary antibody served as negative controls. The slides were developed with an EnVisionTM method (DAKO, Capinteria, CA), visualized using the diaminobenzidine solution, and then lightly counterstained with hematoxylin. Evaluation of immunohistochemical staining reaction was performed in accordance with the immunoreactive score (IRS) proposed by Remmele and Stegner^47^. IRS=SI (staining intensity) × PP (percentage of positive cells). Negative SI=0; weak SI=1; moderate SI=2; strong SI=3. Negative PP=0; 10% PP=1; 11–50% PP=2; 51–80% PP=3; and >80% PP=4. Ten microscopic fields (100 ×) from different areas of each tissue section were used for the IRS evaluation. Slides were examined and scored independently by three pathologists who were blinded to the information of patients.

### Statistical analysis

All data were expressed as means±s.e.m. and were analysed using either one-way analysis of variance or two-tailed unpaired Student's *t*-test. For each parameter of all data presented, *indicates *P*<0.05, **indicates *P*<0.01 and ***indicates *P*<0.005.

### Data availability

The microarray data have been deposited in GEO under the accession codes GSE80065 and GSE80066. Other data that support the findings of this study are available within the article and its Supplementary information files.

## Additional information

**How to cite this article:** Miao, H. *et al.* Macrophage ABHD5 promotes colorectal cancer growth by suppressing spermidine production by SRM. *Nat. Commun.* 7:11716 doi: 10.1038/ncomms11716 (2016).

## Supplementary Material

Supplementary Information Supplementary Figures 1 - 13
