# Supplementary material for: Macrophage ABHD5 promotes colorectal cancer growth by suppressing spermidine production by SRM
Source: Nat Commun. 2016 May 18;7:11716. doi: 10.1038/ncomms11716 (PMC4873969; doi:10.1038/ncomms11716)

Supplementary Figures

Supplementary Figure 1

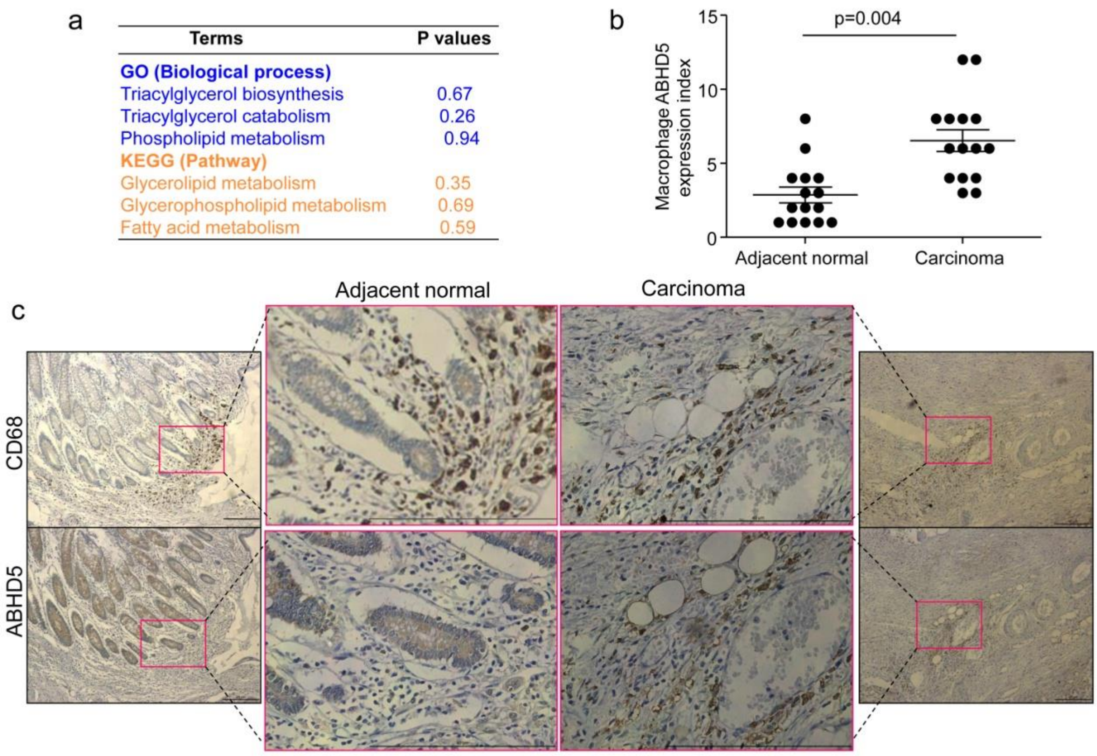

Supplementary Figure 1

Increased ABHD5 expression in human colon cancer associated macrophages.

(a) Murine peritoneal macrophages were treated with regular culture medium (Ctrl) or co-cultured with CRC cells CT-26 (Tumor) for 24 h, and then subjected to gene microarray analysis. The gene microarray data were subjected to GO and KEGG analysis.

(b-c) Expression of macrophage ABHD5 increased in human colon carcinoma tissues. (b) The statistic data was obtained from immunohistochemical staining analysis of 15 samples from 15 patients. Data represent means ± s.e.m., n=15, P=0.004, Student's *t* -test. (c) Representative images from immunohistochemical staining of CD68+ cells (macrophages) in adjacent normal tissue and carcinoma tissue. The corresponding area of infiltrated macrophages was stained with ABHD5 antibody on the consecutive slides. ABHD5 expression in macrophages in tumors was significantly higher than that in adjacent normal tissues. The representative areas of macrophages at higher magnification are displayed in the red square areas. Scale bar represents 100 µm.

Supplementary Figure 2

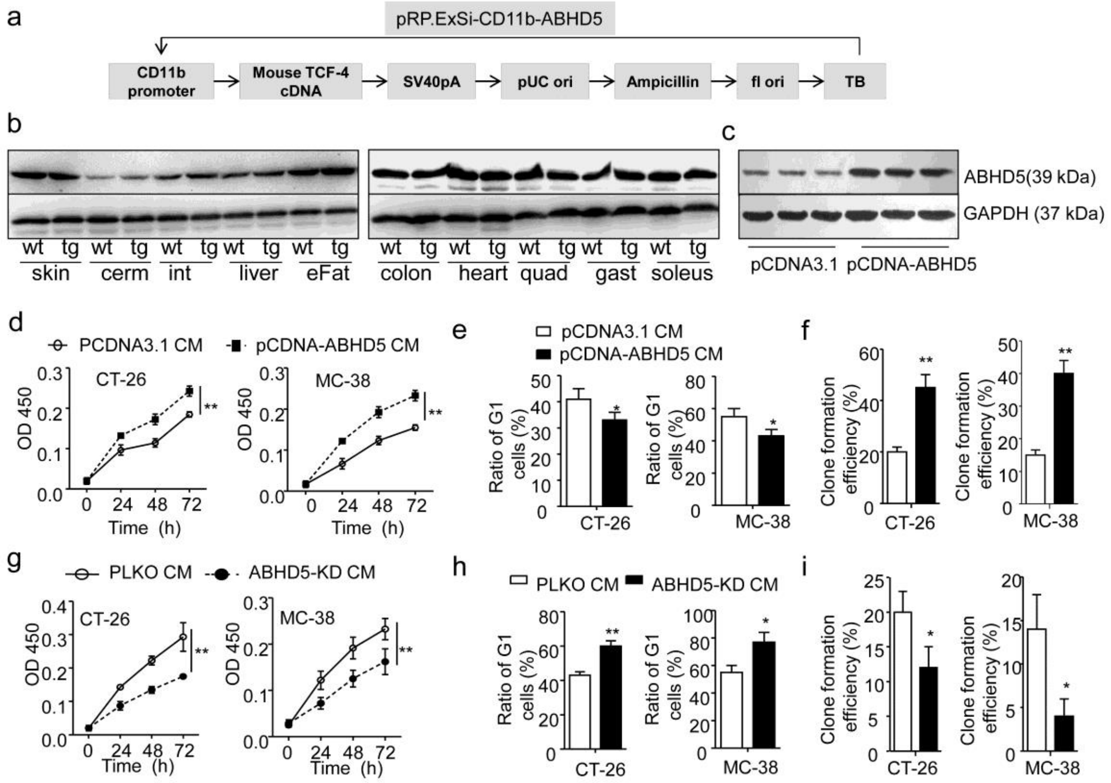

Supplementary Figure 2

Overexpression of CGI-58 in macrophages potentiates colorectal cancer cell growth in vitro

(a) The structure diagram of plasmid used for macrophage specific transgene of ABHD5 (Tg^{ABHD5}).

(b) Immunobloting assay of ABHD5 expression in multiple tissues from 12-week-old male WT or Tg^{ABHD5} mice. cerm, cerebrum; int, intestine; eFat, epididymal fat tissue; quad, quadriceps; gast, gastrocnemius.

(c) Immunobloting assay of ABHD5 expression in pCDNA3.1 or pCDNA3.1-ABHD5-transfected Raw264.7 macrophages.

(d) The colorectal cancer cells CT-26 or MC-38 were treated with conditional medium (CM) from pCDNA3.1 or pCDNA3.1-ABHD5-transfected Raw264.7cells. The cancer cell viability was measured at every 24 hours. (n=5, **p<0.01)

(e) The CT-26 or MC-38 cells were treated with CM from pCDNA3.1 or pCDNA3.1-ABHD5-transfected Raw264.7 cells for 48 h, and the cell cycle was measured by flow cytometry. (n=3, *P<0.05)

(f) The CT-26 or MC-38 cells were treated with CM from pCDNA3.1 or pCDNA3.1-ABHD5-transfected Raw264.7 cells, and the clone formation efficiency was determined. (n=3, **P<0.01)

(g) The CT-26 or MC-38 cells were treated with CM from control (PLKO) or ABHD5-silenced (ABHD5-KD) Raw264.7cells. The cancer cell viability was measured kinetically. (n=5, **p<0.01)

(h) The CT-26 or MC-38 cells were treated with CM from PLKO or ABHD5-KD-Raw264.7 cells for 48 h, and the cell cycle was determined by flow cytometry. (n=3, *P<0.05, **P<0.01)

(i) The CT-26 or MC-38 cells were treated with CM from PLKO or ABHD5-KD-Raw264.7 cells, and the clone formation efficiency was measured. (n=3, *P<0.05)

From panel (d) to (i), the data represent means ± s.e.m, Student's *t* -test.

Supplementary Figure 3

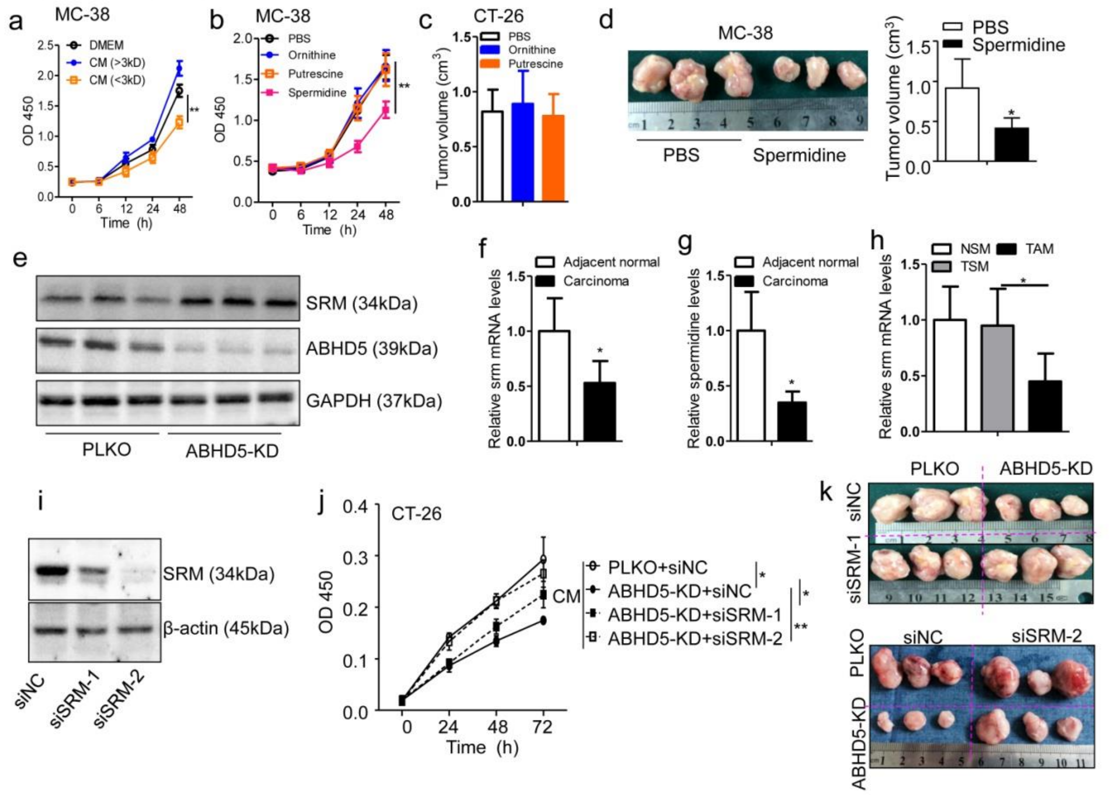

Supplementary Figure 3
Macrophage ABHD5 potentiates colorectal cancer cell growth via SRM-dependent spermidine production.
(a) The CM (<3 kD) from ABHD5-KD RAW cells attenuated the growth of MC-38 cells. The CM of ABHD5-KD RAW cells was separated into two fractions (>3 kD and <3 kD). The cell viability of MC-38 treated with DMEM, CM (>3 kD) or CM (<3 kD) was measured dynamically. The data represent means ± s.e.m., (n=5, **P<0.01, Student's *t* -test). **(b)** The cell viability of MC-38 treated with Ornithine (2 mM), Putrescine (2 mM), Spermidine (2 mM) or PBS as control. The data represent means ± s.e.m., (n=5, **P<0.01, Student's *t* -test). **(c)** The 6-week-old mice were subcutaneously inoculated with CT-26 tumor, and treated with Ornithine (2 mM, 100 μl/2d), Putrescine (2 mM, 100 μl/2d) or vehicle control PBS (100 μl/1d) for 2 weeks. Then, the tumor volume was measured (n=5). **(d)** The 6-week-old mice were subcutaneously inoculated with MC-38 tumor, and treated with spermidine (2 mM, 100 μl/2d) or vehicle control PBS for 2 weeks. Then, the tumor volume was measured, and the representative images were displayed. The data show means ± s.e.m., (n=5, *P<0.05, Student's *t* -test). **(e)** Immunoblotting assays of SRM and ABHD5 in PLKO or ABHD5-KD-macrophages. **(f)** SRM mRNA levels in the macrophages from adjacent normal or carcinoma tissues of human colorectal cancer were measured by Realtime PCR. The data show means ± s.e.m., (n=8, *P<0.05, Student's *t* -test). **(g)** Relative spermidine levels in the macrophages from adjacent normal or carcinoma tissues of human colorectal cancer. The data show means ± s.e.m., (n=8, *P<0.05, Student's *t* -test). **(h)** Macrophages were isolated from the spleen (TSM) and the tumor tissue (TAM) of CT-26 tumor-bearing mice or from the spleen of tumor-free mice (NSM). Then the SRM mRNA levels in macrophages were measured by Realtime PCR. The data show means ± s.e.m., (n=6-8, *P<0.05, Student's *t* -test) **(i)** Immunoblotting assay of SRM in macrophages transfected with control siRNA (20 nmol/ml), SRM specific siRNA1 (20 nmol/ml) or siRNA2 (20 nmol/ml). **(j)** Viability of CT-26 cells treated with different CM from PLKO or ABHD5-KD-Raw264.7 macrophages treated with control siRNA (20 nmol/ml), SRM siRNA1 (20 nmol/ml) or siRNA2 (20 nmol/ml). The data show means ± s.e.m., (n=5, *P<0.05 **P<0.01, Student's *t* -test). **(k)** The 6-week old mice were subcutaneously inoculated with CT-26 tumor, and treated with the CM as described in **(j)** for 2 weeks. The subcutaneous tumors were dissected and representative images were displayed.

Supplementary Figure 4

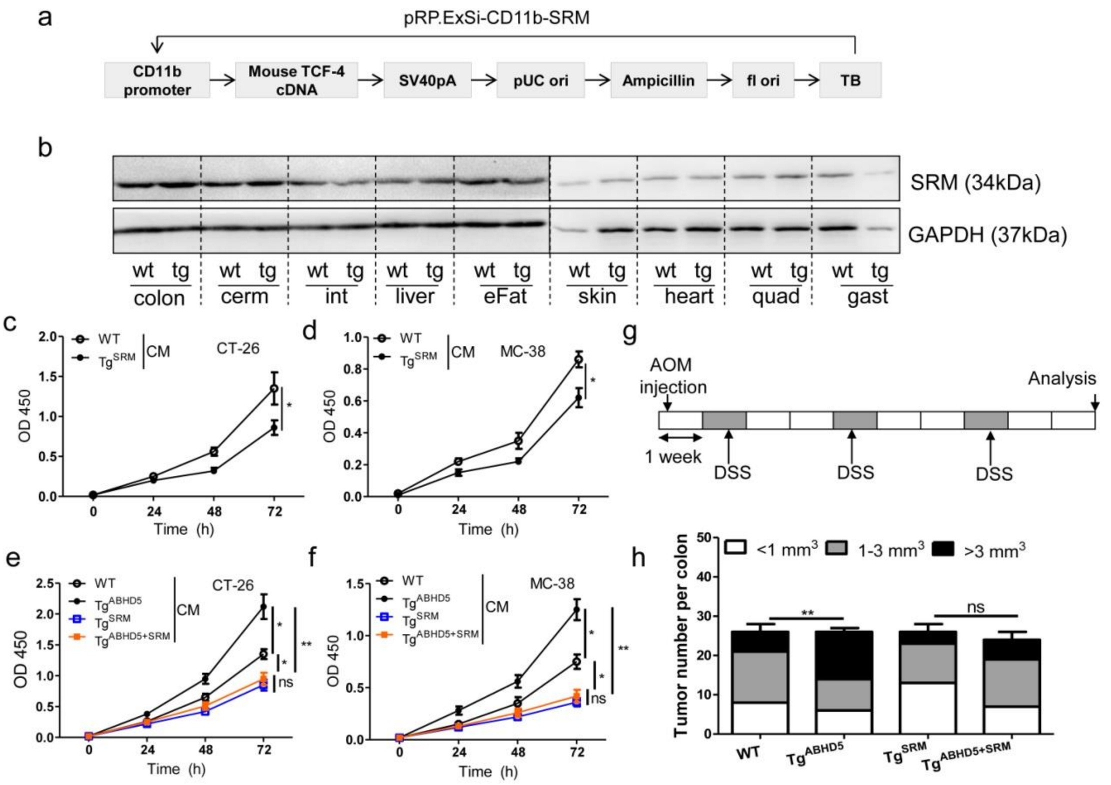

Supplementary Figure 4

Macrophage SRM prevents ABHD5-potentiated colorectal cancer growth.

(a) The structure diagram of plasmid used for macrophage specific transgene of SRM (Tg^{SRM}).

(b) Identification of macrophage specific transgene of *SRM* gene. Immunoblotting assay of SRM expression in multiple tissues and organs in 12-week-old male mice. cerm, cerebrum; int, intestine; eFat, epididymal fat tissue; quad, quadriceps; gast, gastrocnemius.

(c,d) The cell viability of CT-26 **(c)** and MC-38 **(d)** treated with CM from peritoneal macrophages of WT or Tg^{SRM} mice. The data represent means ± s.e.m., (n=5, *P<0.05, Student's *t* -test).

(e, f) The cell viability of CT-26 **(e)** and MC-38 **(f)** treated with CM from peritoneal macrophages of WT, Tg^{ABHD5}, Tg^{SRM} and $Tg^{ABHD5+SRM}$ mice. The data show means ± s.e.m., (n=5, *P<0.05, **P<0.01, Student's *t* -test). ns, not significant

(g) Schedule diagram of azoxymethane (AOM) and dextran sulfate soldium (DSS)-induced colorectal cancer.

(h) The 8-week-old WT, Tg^{ABHD5}, Tg^{SRM} and $Tg^{ABHD5+SRM}$ mice were treated according to the program described in **(g)**, and then the intestinal tumors in each group was evaluated by volume and numbers. The data show means ± s.e.m., (n=6, **P<0.01, Student's *t* -test). ns, not significant

Supplementary Figure 5

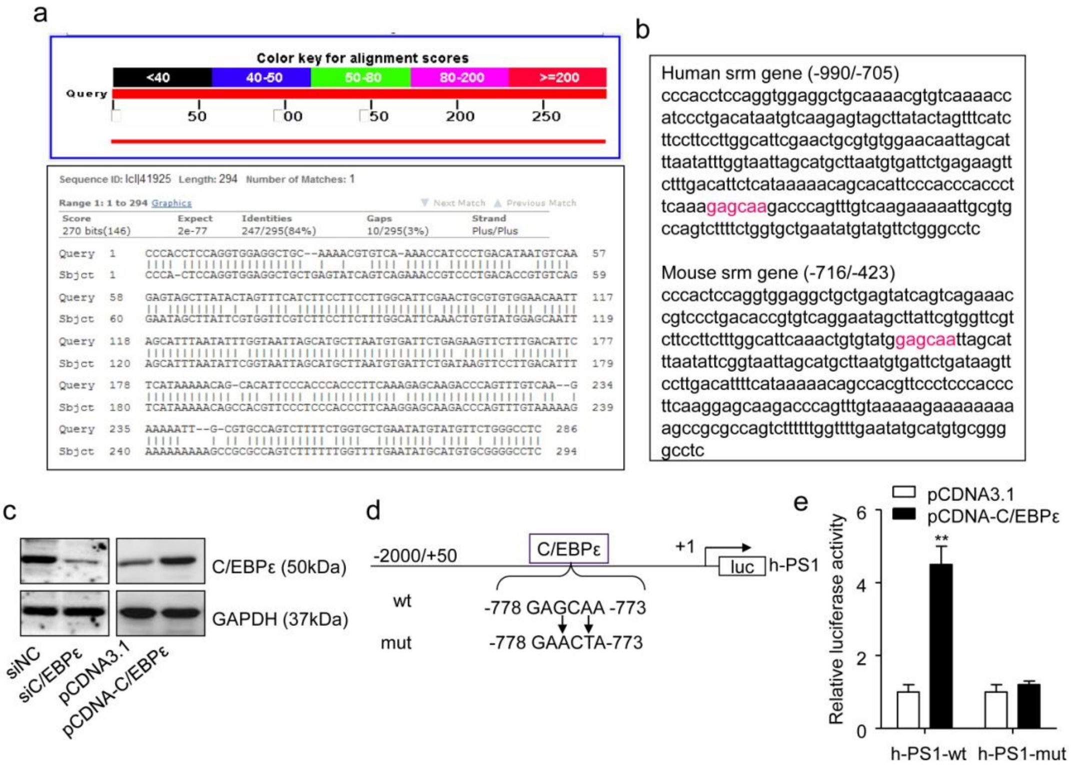

Supplementary Figure 5

C/EBPε potentiates human SRM transcription by binding to a C/EBPε element locating at -778/-773 in human SRM promoter.

(a) A highly conserved promoter region was found by aligning the promoter sequences of human and mouse *srm* genes.

(b) The detailed sequences of the conserved promoter region in human and mouse *srm* genes.

(c) Immunoblotting assay of C/EBPε in the Raw264.7 cells transfected with siNC (20 nmol/ml), si/C/EBPε (20 nmol/ml), pCDNA3.1 (0.4 μg/ml) or pCDNA-C/EBPε (0.4 μg/ml) for 24 h.

(d) Promoter sequences -2000/+50 in human *SRM* gene were subcloned into PGL4-basic vector to construct h-PS1. A C/EBPε binding element locating at -778/-733 was mutated from GAGCAA (h-PS1-wt) to GAACTA (h-PS1-mut).

(e) The Raw264.7 cells were cotransfected with reporter construct h-PS1-wt (0.4 μg/ml) or h-PS1-mut (0.4 μg/ml) plus pCDNA3.1 (0.4 μg/ml) or pCDNA-C/EBPε (0.4 μg/ml) for 24 h, and then reporter gene assay was performed. The data show means ± s.e.m., (n=3, **P<0.01, Student's *t* -test).

Supplementary Figure 6

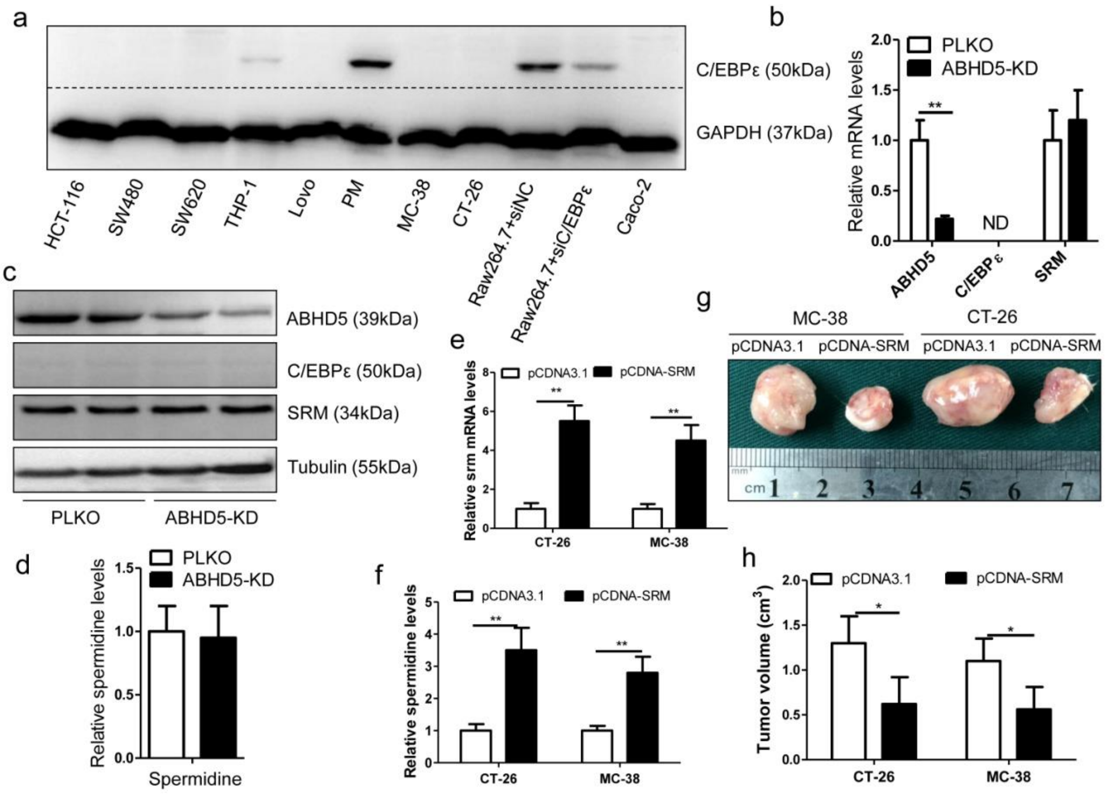

Supplementary Figure 6

ABHD5 doesn't regulate SRM expression in colorectal cancer cells.

(a) Immunoblotting assay of C/EBPε in different colorectal cancer cells and macrophages.

(b) Relative mRNA levels of ABHD5, C/EBPε and SRM in the ABHD5-silenced or control HCT-116 cells. Histograms show means ± s.e.m., (n=3, **p<0.01, Student's *t* -test). ND, not detectable

(c) Immunoblotting assay of ABHD5, C/EBPε and SRM in the PLKO control or ABHD5-KD HCT-116 cells.

(d) Relative levels of spermidine in the control or ABHD5-KD HCT-116 cells. The data show means ± s.e.m., (n=4, Student's *t* -test).

(e, f) Relative levels of SRM mRNA **(e)** and spermidine **(f)** in MC-38 and CT-26 cells which were stably transfected with pCDNA3.1 or pCDNA-SRM. The data show means ± s.e.m., (n=4, **p<0.01, Student's *t* -test).

(g, h) The pCDNA3.1 or pCDNA-SRM-transfected MC-38 or CT-26 cells were subcutaneously inoculated (10^6 cells in 100 μl) in the 6-week old mice and the tumor volume was measured 14 days after tumor cell injection **(h)**. Representative tumor images were displayed **(g)**. Histograms show means ± s.e.m., (n=5, *p<0.05, Student's *t* -test).

Supplementary Figure 7

Uncropped gel images for Figure 1-2

Figure 1d

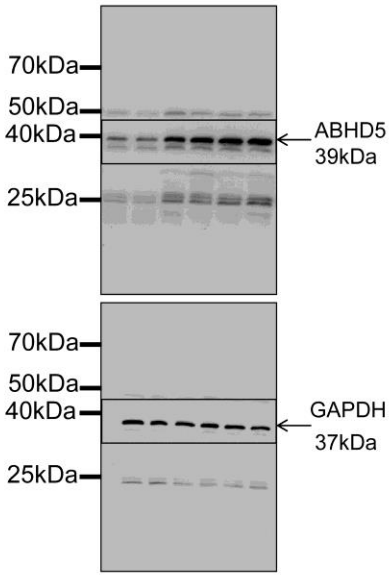

Figure 1g

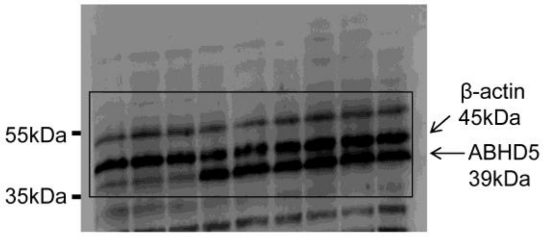

Figure 2b

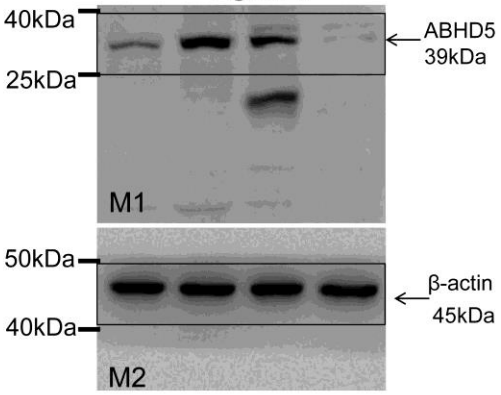

Supplementary Figure 8

Uncropped gel images for Figure 4-5

Figure 4a

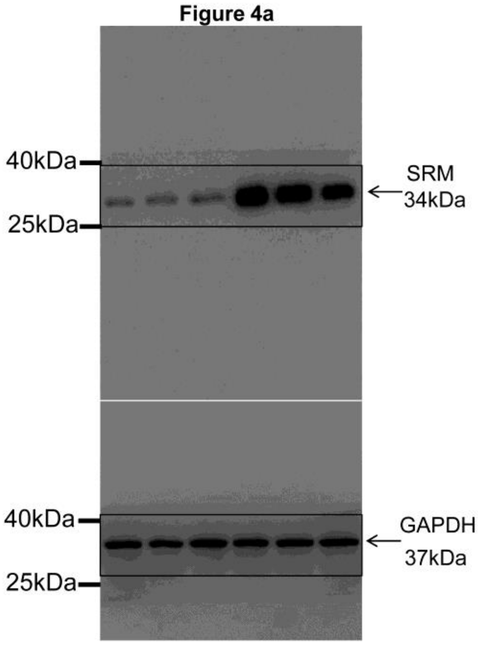

Figure 5h

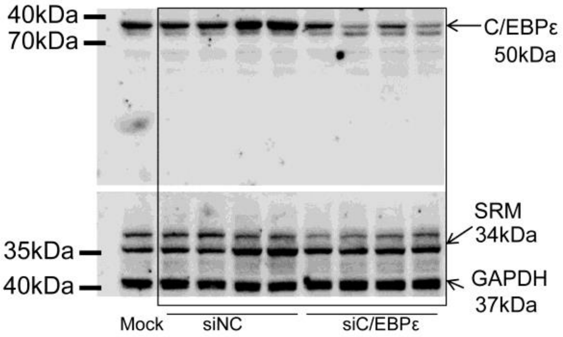

Supplementary Figure 9

Uncropped gel images for Figure 6

Figure 6d

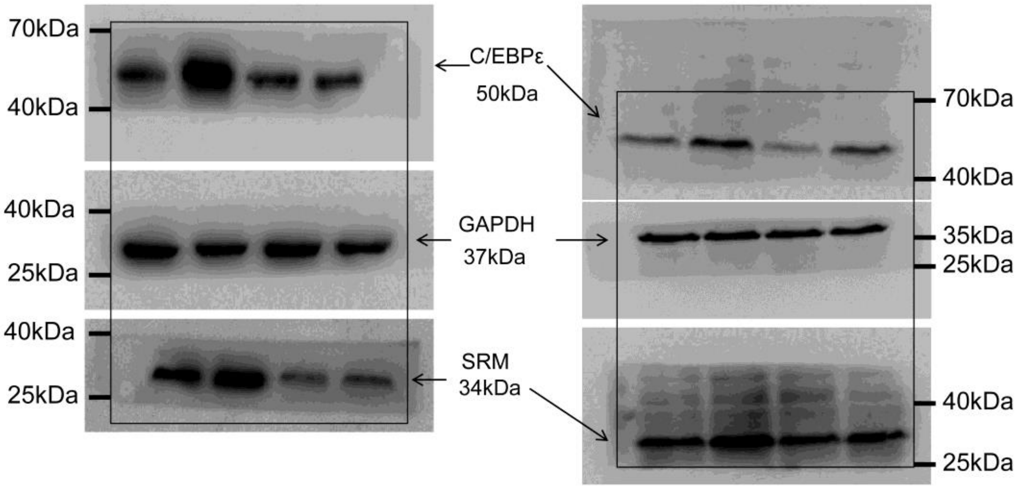

Supplementary Figure 10

Uncropped gel images for Figure S2

Figure S2b

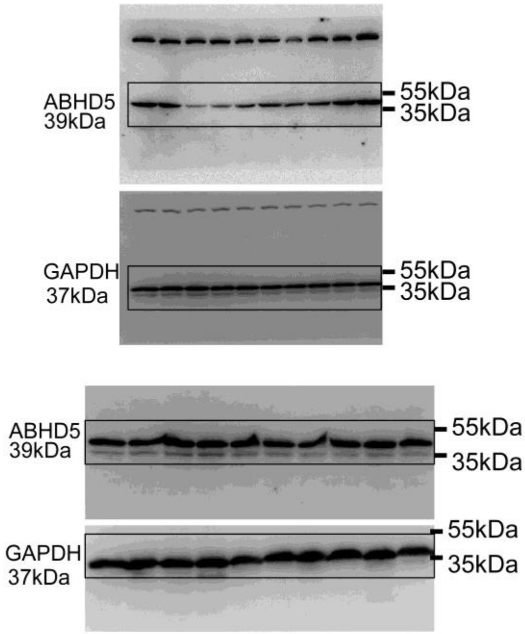

Figure S2c

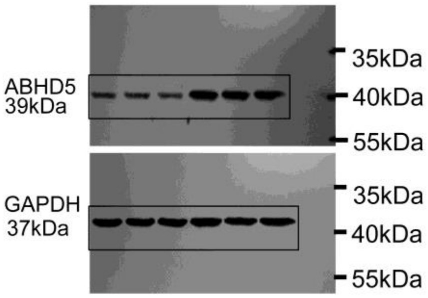

Supplementary Figure 11

Uncropped gel images for Figure S3

Figure S3e

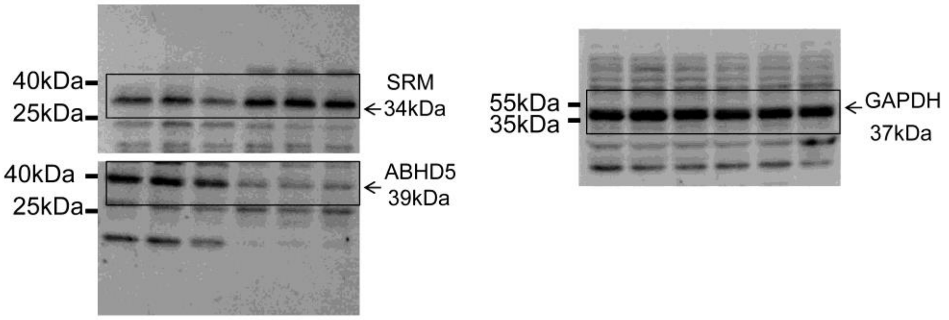

Figure S3i

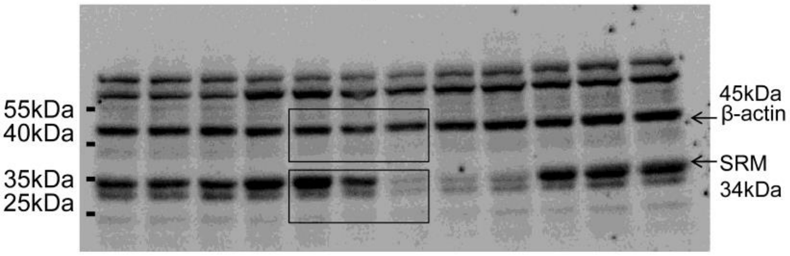

Supplementary Figure 12

Uncropped gel images for Figure S4-5

Figure S4b

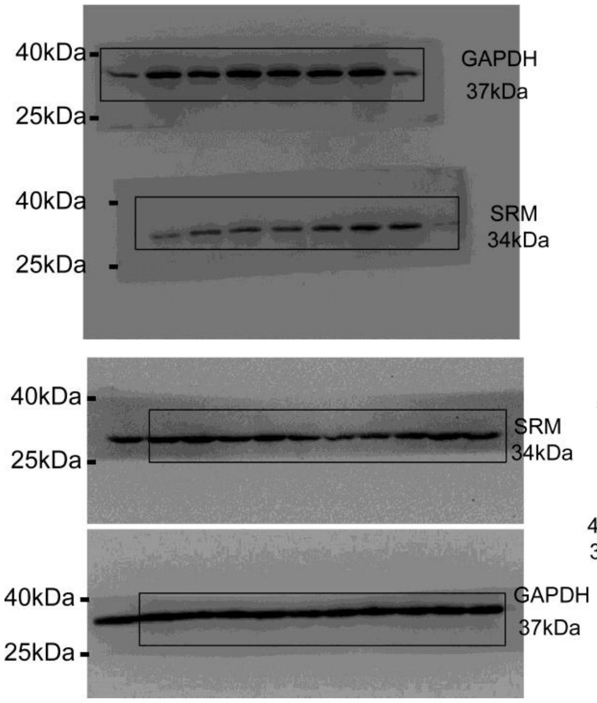

Figure S5c

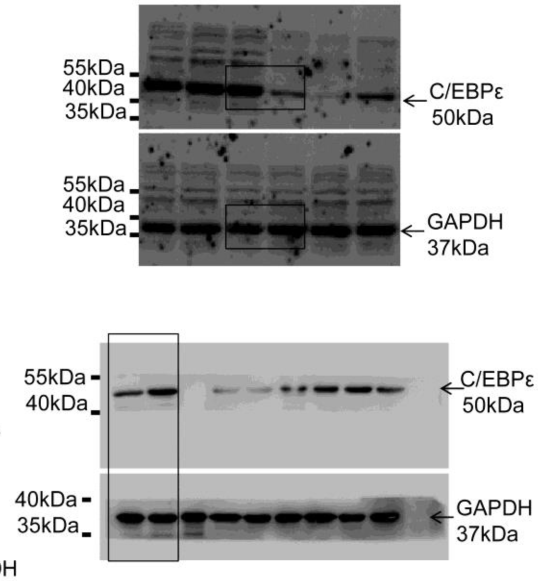

Supplementary Figure 13

Uncropped gel images for Figure S6

Figure S6a

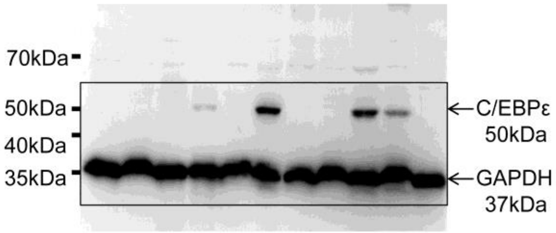

Figure S6c

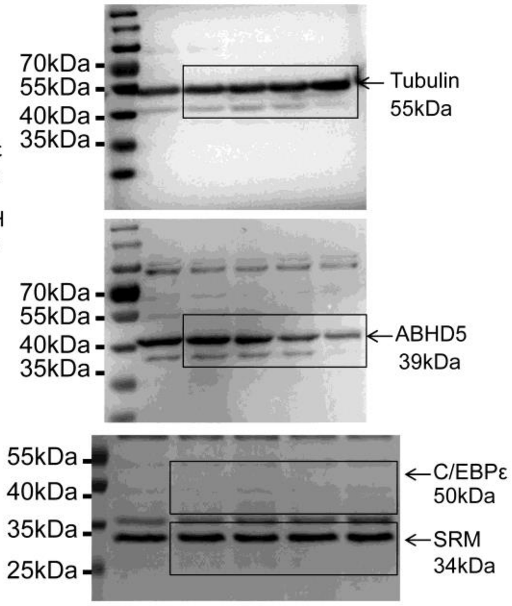

Supplement: Supplementary Information — Supplementary Figures 1 - 13 [file ncomms11716-s1.pdf]
